# Modulating Drought Stress Response of Maize by a Synthetic Bacterial Community

**DOI:** 10.3389/fmicb.2021.747541

**Published:** 2021-10-21

**Authors:** Jaderson Silveira Leite Armanhi, Rafael Soares Correa de Souza, Bárbara Bort Biazotti, Juliana Erika de Carvalho Teixeira Yassitepe, Paulo Arruda

**Affiliations:** ^1^Centro de Biologia Molecular e Engenharia Genética, Universidade Estadual de Campinas (UNICAMP), Campinas, Brazil; ^2^Genomics for Climate Change Research Center (GCCRC), Universidade Estadual de Campinas (UNICAMP), Campinas, Brazil; ^3^Departamento de Genética e Evolução, Instituto de Biologia, Universidade Estadual de Campinas (UNICAMP), Campinas, Brazil; ^4^Embrapa Informática Agropecuária, Campinas, Brazil

**Keywords:** SynCom, plant microbiome, plant phenotyping, drought stress, maize, plant growth-promoting, PGP, synthetic microbial community

## Abstract

Plant perception and responses to environmental stresses are known to encompass a complex set of mechanisms in which the microbiome is involved. Knowledge about plant physiological responses is therefore critical for understanding the contribution of the microbiome to plant resilience. However, as plant growth is a dynamic process, a major hurdle is to find appropriate tools to effectively measure temporal variations of different plant physiological parameters. Here, we used a non-invasive real-time phenotyping platform in a one-to-one (plant–sensors) set up to investigate the impact of a synthetic community (SynCom) harboring plant-beneficial bacteria on the physiology and response of three commercial maize hybrids to drought stress (DS). SynCom inoculation significantly reduced yield loss and modulated vital physiological traits. SynCom-inoculated plants displayed lower leaf temperature, reduced turgor loss under severe DS and a faster recovery upon rehydration, likely as a result of sap flow modulation and better water usage. Microbiome profiling revealed that SynCom bacterial members were able to robustly colonize mature plants and recruit soil/seed-borne beneficial microbes. The high-resolution temporal data allowed us to record instant plant responses to daily environmental fluctuations, thus revealing the impact of the microbiome in modulating maize physiology, resilience to drought, and crop productivity.

## Introduction

Crop plants are continuously challenged by adverse environmental conditions that can severely impact their productivity. As sessile organisms, plants have evolved genetically encoded mechanisms to efficiently thrive in adverse circumstances. Breeders have explored the genetic variability associated with tolerance against drought and heat stresses, which are among the most limiting factors for crop production (Lesk et al., 2016). However, genetically encoded water usage traits alone may not be sufficient to make plants better adapt to water restriction and high temperature conditions. Microbial communities associated with plant roots, stems and leaves have also been shown to play a fundamental role in shaping plant responses to biotic and abiotic stresses and modulating plant phenotypic plasticity (Compant et al., 2019; Vannier et al., 2019; Beirinckx et al., 2020; Chai et al., 2021).

Plants and their associated microbiome have coevolved over a million years under adverse environmental conditions. During this process, evolution may have favored plants recruiting microbial communities that positively affected their fitness by providing or modulating beneficial functions related to phytohormone balance, plant adaptation to drought, nutrition uptake, and disease suppression (Zilber-Rosenberg and Rosenberg, 2008; Lemanceau et al., 2017; Chaudhry et al., 2021). This scenario implies that plants cannot be studied as isolated entities but rather as a unit formed by the plant and its associated microbiome, the holobiont (Vandenkoornhuyse et al., 2015). However, with the exception of few cases, such as nitrogen fixation and phytohormones production (Carvalho et al., 2014), there is little knowledge regarding other modes by which microbes can influence plant phenotypic plasticity.

Investigating how plant–microbe interactions affect plant responses and physiology requires multidisciplinary approaches that allow the integration of different types of data from both the plant and its microbiome (de Souza et al., 2020). The broad concept of plant phenotyping implies the understanding of plant physiological, morphological and biochemical status by methods capable of quantifying relevant plant traits. An increased number of phenotyping platforms have been designed, reducing both time and costs by automating plant cultivation and assessment (Fiorani and Schurr, 2013). However, as plant growth is a dynamic process, common phenotyping strategies lack the ability to monitor plant physiological parameters in real time (Halperin et al., 2017; Rouphael et al., 2018). In most cases, these platforms rely on limited frequency of measures or unsynchronized data points as plants are evaluated individually and during distinct periods. A comprehensive physiological evaluation demands a continuous time series to acquire a detailed physiological profile of each individual plant at the same time points.

We have previously shown that a synthetic community (SynCom) composed of naturally occurring, highly abundant bacteria from the sugarcane root and stalk core microbiomes increased biomass and enhanced root system development of early-stage maize (*Zea mays* L.) plants (de Souza et al., 2016; Armanhi et al., 2018). Here, we investigated the impact of this SynCom on the physiological behavior and yield of mature maize plants under drought conditions. We developed an automated and non-invasive real-time phenotyping platform to evaluate both plant performance and physiological responses in a one-to-one (plant–sensors) set up throughout the entire plant life cycle. The continuous monitoring of individual plants produced high-quality data and images with resolution sufficient to inspect small variations in plant physiological parameters of biological relevance. SynCom was found to modulate plant leaf temperature (T_*leaf*_) and water usage with significant differences among plant genotypes. The results were discussed in the context of expanding our comprehension of crop functional trait responses for the mitigation of environmental stresses toward developing microbiome technologies for agricultural sustainability.

## Materials and Methods

### Plant Material, Experimental Conditions and Inoculation

Seeds of the three commercial maize hybrids DKB177 PRO3 (Monsanto, Brazil), SX7341 VIP3 (Syngenta, Brazil), and P3707VYH (DuPont Pioneer, Brazil) were purchased and stored at 4°C prior to sowing. These hybrids were chosen because of their high yield potential and for being locally adopted by farmers when the experiment was set up. The experiments were installed in a 90-m^2^ netted greenhouse facility of the School of Agricultural Engineering at the University of Campinas (22°49′11.94″ S 47°3′40.96″ W) under natural environmental conditions from late August to mid-December 2018. The experimental period was counted as days after sowing (DAS) starting on the day seeds were sown (0 DAS) to plant harvesting (117 DAS). From 1 to 65 DAS, the photoperiod was extended until 8:00 pm with halogen bulbs PAR38 100 W (FLC, Brazil) at a density of 1 bulb m^–2^. Plants were grown in 18-L pots filled with a commercial substrate (Biogrow, Brazil) modified to contain a 7:1 sphagnum:perlite mixture (Agrolink, Brazil). Pots were fertilized with PG MIX 14-16-18 (Yara, Norway) and Osmocote 15-9-12 (ICL Specialty Fertilizers, Summerville, SC, United States) before planting according to suppliers’ recommendations for maize. Three seeds were sown per pot, but only the best developed seedling was kept after 9 DAS. A total of 432 pots were distributed along the greenhouse in a twin row experimental design (Supplementary Figure 1) and irrigated once a day (at 8:20 am) with ∼430 mL of water by an automated piped system settled at 72 mL min^–1^ from 0 to 56 DAS and three times a day (at 8:20 am, 10:20 am, and 6:20 pm) with the same volume of water each time from 57 to 117 DAS. Plants were subjected to well watering (WW) or drought stress (DS) conditions. WW-treated plants received the full irrigation schedule during all experiments, unless otherwise mentioned. On cloudy days, irrigation was reduced to half the volume to avoid overwatering plants. DS-treated plants received full irrigation until 49 DAS and were then subjected to a 50% WW regime (50–52 DAS), followed by 25% WW (53–80 DAS). Plants were at the V10–V11 stage when exposed to DS. Plants under DS were then rehydrated using the full irrigation regime in the early evening at 80 DAS. Due to occasional rain that increased air relative humidity (RH) to 100%, irrigation was suspended from 61 to 65 DAS and reduced to half from 88 to 108 DAS for all treatments. Plants used for time-lapse imaging were grown under the same abovementioned conditions and exposed to severe drought stress (SDS) (complete water withdrawal from 50 to 84 DAS), with rehydration performed at 84 DAS until full recovery.

In this work, we used a SynCom assembled by mixing naturally occurring, highly abundant bacterial strains from the sugarcane root and stalk core microbiomes that were shown to robustly colonize maize plants (de Souza et al., 2016; Armanhi et al., 2018). As previously described, the SynCom was prepared by individually growing 17 community-based isolates from the sugarcane Community-Based Culture Collection (CBC) in liquid culture media to late exponential phase and pooled in equal concentration based on optical density (OD) (Armanhi et al., 2018). Bacterial pellets were washed and resuspended in 0.1× Hoagland’s solution to reach the final OD_620__nm_ of 0.6. Seeds of the three commercial maize hybrids were soaked in SynCom solution for 3 h prior to sowing. Uninoculated seeds were soaked in sterile 0.1× Hoagland’s solution. After planting, 1 mL of the SynCom solution was pipetted over each seed in each individual pot, while uninoculated seeds received the same volume of sterile 0.1× Hoagland’s solution.

The number of emerged seedlings was counted daily in every pot from 3 to 7 DAS. In mature plants, days to anthesis and to silking were considered at the first sign of shedding pollen and extruding silks from the husk, respectively, as observed daily from 70 to 86 DAS. Statistical analyses of seedling emergence timing and reproductive stages of plants were performed by unpaired *t*-test. The flowering time was registered for plants with both anthesis and silking, and plants that lacked pollen shedding (hidden tassel in the whorl or not extruded anthers) or silk extrusion were considered flowerless. The effect of SynCom inoculation on the total number of flowering plants was considered significant when the number of flowering plants was exceeded by 10% the number of flowerless plants.

### Plant Sampling, DNA Extraction and 16S rRNA Gene Sequencing for Microbial Profiling

Microbial profiling was performed as previously described (de Souza et al., 2016; Armanhi et al., 2018), with bacterial communities associated with plant roots assessed through 16S profiling using roots sampled from 4 plants (biological replications) per treatment harvested at stages V11–V12 (53 DAS). Plant roots were cleaned of soil excess by hand shaking, then frozen in liquid nitrogen. Cryopreserved samples were ground under cryogenic conditions in a stirred bead mill. DNA of powdered samples was extracted, and V4 16S regions were amplified. Libraries were sequenced in a MiSeq sequencing platform using reagent kit v3 in a 2 × 300 run (Illumina, San Diego, CA, United States). Bacterial taxonomic assignment was performed using the software SYNTAX (Edgar, 2016) and the SILVA v123 database (Quast et al., 2013). Enrichment and depletion of microbial groups were assessed by comparing the differential relative abundance of each operational taxonomic unit (OTU) between microbial profiles of inoculated and uninoculated with a Kruskal–Wallis test with *P* < 0.05. The same test was performed to detect robustness of colonization by comparing the abundance of SynCom OTUs in inoculated and uninoculated plants using a data analysis pipeline previously described (Armanhi et al., 2018).

### Harvesting and Assessment of Plant Traits

Apart from plants sampled for microbial profiling, the remaining 10 plants per treatment were harvested at maturity at 117 DAS. For plant biomass estimation, the aerial parts of individual plants and ears were dried at 65°C for 7 days. Kernels were manually removed from the cob, counted and weighed. Yield per plant was considered the weight of grains per plant. The harvest index (HI), in %, was considered as follows:


$$\text{HI}=\frac{\mathrm{mean}\mathrm{grain}\mathrm{yield}}{\mathrm{mean}\mathrm{total}\mathrm{aboveground}\mathrm{biomass}}$$

where both mean grain yield and mean total aboveground biomass are expressed in grams (Hütsch and Schubert, 2018). All collected data from individual inoculated and uninoculated plants were evaluated for normality by the Shapiro–Wilk test and for homogeneity of variances by Levene’s test. The statistical significance of the phenotypic values was determined for all parameters using a three-way ANOVA with the following factors: genotype, irrigation regime, inoculation and all their possible interactions. Mean values were compared when significant factors or interactions were observed using Tukey’s test. Statistical analyses were performed using RStudio v4.0.3 (RStudio Team, 2020) and the package “agricolae” (de Mendiburu, 2020).

### Monitoring of Environmental Conditions

Environmental conditions were collected by four stations placed along the greenhouse. Air temperature (°C) and RH (%) were measured using DHT22 sensors (Adafruit, New York, NY, United States), while light intensity was captured through BH1750FVI sensors (Adafruit, New York, NY, United States) (Supplementary Figures 2A,B). DHT22 was placed on fixed stations 3 m above the ground, while BH1750FVI was placed on moving platforms vertically adjusted at the same level as the plant canopy (Supplementary Figure 2C). Light intensity was collected in lux and converted to photosynthetically active radiation (PAR) (μmol m^–2^ s^–1^) using the correction factor of 0.0185×. Data from environmental sensors were taken by each station every 15 min and stored in a data logger for further analyses. Vapor-pressure deficit (VPD) (kPa) was determined for each data point using the Arden–Buck equation (Buck, 1981), as follows:


$$\text{VPD}=\left(1-\frac{\text{RH}}{100}\right)\times 0.61121\times \text{exp}\left(\frac{17.502\times \text{T}}{240.97+\text{T}}\right)$$

where T is the air temperature (°C) and RH is the air relative humidity (0–100%). In downstream analyses, data points of all environmental variables were grouped every 30 min, and average values were calculated considering data from all four stations. Outliers were removed based on Tukey’s interquartile range (IQR) method.

### Monitoring of Plant Physiological Parameters

Forty-eight plants were individually equipped with a set of sensors for T_*leaf*_, sap flow and soil water content (SWC). Data were collected for all monitored plants every 5 min and stored in individual data loggers prepared for each plant. T_*leaf*_ was measured using a 10 K-ohm thermistor placed in the center of a 2×2-cm squared 1-mm-thick cork. The sensor was placed onto the abaxial surface of the 8th leaf of plants 15 cm from the leaf collar (Supplementary Figures 2D,E). Xylem sap flow was measured following the heat dissipation method (Granier, 1987) (Supplementary Figure 2F). The water flow upward in plant stalks was considered sap flow. SWC was measured through capacitive soil moisture sensors v1.2 (Adafruit, New York, NY, United States) (Supplementary Figure 2G). Data points from each individual sensor type were further rounded every 30 min, in accordance with the environmental data, with averages calculated and outliers removed from the four biological replicates.

### Connectivity, Data Retrieval, Automated Image Capture and Time-Lapse Movie

Data from all environmental and physiological parameter sensors were automatically collected by Arduino Uno boards (Arduino, Italy) gathered in a Raspberry Pi 3 model B+ microcontroller (Raspberry Pi Foundation, United Kingdom) using custom scripts written in Python for data communication. Images were automatically captured by custom scripts run in Raspberry Pi. High-resolution RGB images were taken using a 20-MP digital camera Coolpix S3700 (Nikon, Japan) every 10 min during all experiments. The frequency of image capture was increased to every 3 min during DS and every 2 min during plant recovery to maximize the resolution of analysis during these periods. Two cameras were simultaneously allocated to record images for the time-lapse movie and the entire experimental setup. A time-lapse movie was generated using the open-source multimedia software FFmpeg^1^.

### Data Analysis, Graphs and Figures

All data points collected from sensors were analyzed in a processing pipeline written in Python v3.7.1 run in Jupyter notebook v4.4.0 (Kluyver et al., 2016). Data management and mathematical functions were performed using Pandas v0.23.4 (McKinney and Sheaffer-Jones, 2010), NumPy v1.15.4 (van der Walt et al., 2011), and SciPy v1.1.0 (Jones et al., 2001) libraries. Figures 3–5, and Supplementary Figures 6A, 7A–C, 8–10 were prepared using the Matplotlib v3.0.2 (Hunter, 2007) library. Figures 2, 6A,B, and Supplementary Figures 4, 5, 11 were drawn in GraphPad Prism v8.2.0 (GraphPad Software, San Diego, CA, United States)^2^. All figures were prepared with the effective use of colors to help people with low visual acuity or color blindness.

## Results

### Time-Lapse Imaging Records Differences in Plant Behavior Induced by Synthetic Community Inoculation in Commercial Maize Hybrids Under Severe Drought

A SynCom assembled with bacterial strains highly abundant in sugarcane root and stalk core microbiomes was previously shown to robustly colonize plant organs, improve root architecture and increase the biomass of young maize plants (Armanhi et al., 2018; de Souza et al., 2019). Here, we asked whether this SynCom also elicits drought tolerance in mature maize plants. For this purpose, we set an experiment to evaluate whether there were differences between SynCom-inoculated and uninoculated plants under SDS. Three commercial maize hybrids (DKB177, SX7341, and P3707VYH) were grown under a regular watering regime until 50 DAS, from which they were subjected to complete water withdrawal for 34 days, followed by rehydration. To closely observe the response of each hybrid, plants were monitored by high-resolution RGB imaging every 3 min during SDS and every 2 min during rehydration (Supplementary Movie 1).

**FIGURE 1:**
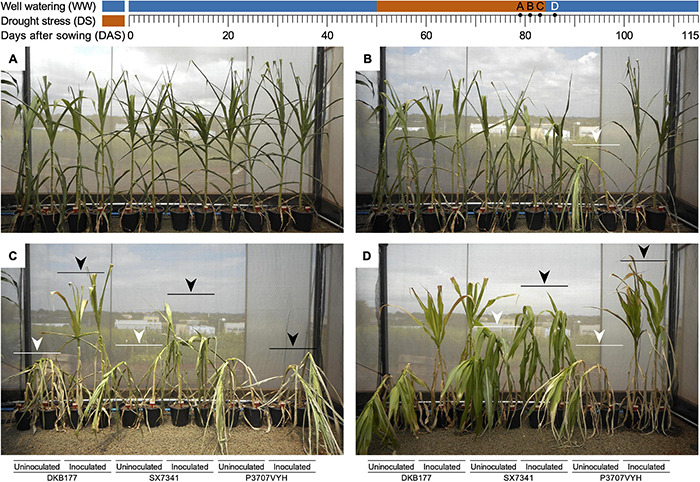
A SynCom containing beneficial microbes induces a physiological response against DS in three commercial maize hybrids. **(A)** Plants kept in SDS for 29 days (79 DAS) had their leaves rolled inward, and older leaves fell for all hybrids, regardless of whether they were inoculated. **(B)** P3707VYH was the less tolerant hybrid in the absence of SynCom, completely bent after 31 days of SDS (81 DAS), in contrast to the inoculated hybrid (white arrow). **(C)** Uninoculated DKB177 and SX7341 were completely bent (83 DAS), as shown by the white arrows. In the presence of SynCom, plants were maintained in a straight position (DKB177) and partially or completely bent (SX7341 and P3707VYH, respectively), as shown by the black arrows. **(D)** Inoculated plants (SX7341 and particularly P3707VYH) straightened 2 days after rewatering (86 DAS; black arrows), while uninoculated plants were not capable of completely recovering their structure (white arrows). Detailed results are shown in Supplementary Figure 3 and Supplementary Movie 1. WW, well watering; DS, drought stress; DAS, days after sowing; SDS, severe drought stress.

All plants, inoculated or not, displayed leaf rolling and unrolling in response to daily temperature and RH changes. After 26 days of SDS (76 DAS), leaf rolling movements increased in frequency, and leaves were maintained permanently rolled inward for SX7341 and P3707VYH (Supplementary Figures 3A,B). After 28 days of SDS (78 DAS), all hybrids exhibited strong signals of stress characterized by leaf bending, a symptom that intensified over the following days (Figure 1A and Supplementary Figure 3C).

In general, under SDS, the stalks of uninoculated plants bent before those of inoculated plants. The uninoculated P3707VYH plants displayed the first bending response, starting after 30 days of SDS (80 DAS) and being completely bent at 81 DAS (Figure 1B and Supplementary Figure 3D). The stalks of the uninoculated DKB177 and SX7341 plants were completely bent by 83 DAS (Figure 1C and Supplementary Figures 3E,F). SynCom inoculation delayed stalk bending in P3707VYH by 1 day (Supplementary Figure 3D). Interestingly, the inoculated SX7341 plants did not show a clear difference in the timing of stalk bending when compared with uninoculated plants, although the effect of SDS was less severe in SynCom-inoculated plants (Figure 1C and Supplementary Figure 3F). At later stages of SDS treatment (34 days of SDS; 84 DAS), all uninoculated plants bent as a response to turgor loss due to severe water deficit. The inoculated P3707VYH plants completely bent at 34 days of SDS, while the inoculated SX7341 plants partially bent. Interestingly, the inoculated DKB177 plants did not bend even at the most severe stage of water deficit (Supplementary Figure 3G).

After rewatering at 84 DAS, the inoculated SX7341 plants immediately recovered and straightened out, an effect only observed 1 day later for their uninoculated counterpart. Similar behavior was found for the P3707VYH plants, although inoculated plants were only completely recovered from bending 2 days after rewatering (86 DAS). Nevertheless, uninoculated SX7341 and P3707VYH plants were unable to completely recover upon rehydration, as their stalks remained partially bent. The hybrid DKB177 was the most responsive to inoculation. Despite the fact that the leaves of both inoculated and uninoculated DKB177 plants remained greener and unrolled, rehydration had no effect on recovering the turgor of the uninoculated plants. In contrast, the inoculated DKB177 plants remained completely straightened during SDS and recovery (Figure 1D and Supplementary Figure 3H). The effect of SDS and recovery upon rehydration can be seen in the time-lapse video recorded from the late stage of water restriction until complete recovery after rewatering (Supplementary Movie 1).

### Synthetic Community Inoculation Reduces the Yield Loss of Commercial Maize Hybrids Under Drought Stress

To better understand the mechanisms underlying plant growth promotion and abiotic stress tolerance induced by SynCom inoculation, we designed a second experiment to dissect the physiological parameters, yield components and colonization patterns of maize hybrids under DS (Supplementary Figure 1). The plants subjected to DS were well watered until 50 DAS, from which they were subjected to a reduced irrigation regime until 80 DAS. We intentionally applied DS in early reproductive stages when plants were most susceptible to drought.

Overall, the germination rate was not affected by SynCom inoculation, except for DKB177, which showed a slight reduction in the number of emerged seedlings per pot. Among all three hybrids, only SX7341 presented uniformity in seedling emergence whether inoculated or not. Particularly for P3707VYH, SynCom inoculation delayed seedling emergence without reducing the total number of emerged seedlings (Supplementary Figures 4A–C). The anthesis occurred from 70 to 86 DAS. Both anthesis and silking were delayed by DS, with inoculation having no significant effect on synchrony between flowering times. Pollen shedding started at 70–76 DAS under WW and 80–85 DAS under DS conditions, with an average delay of 9 days regardless of inoculation. DS-treated plants also delayed silking by 8 days, on average, with silks extruded from 71 to 78 and 81 to 86 DAS for WW- and DS-treated plants, respectively (Supplementary Figures 4D,E). No significant differences in anthesis-silking interval (ASI) between inoculated and uninoculated plants were observed. Although few plants did not shed pollen or did not extrude silks, these characteristics were not influenced by SynCom inoculation under WW conditions. However, the percentage of plants flowering during DS increased, for inoculated DKB177 at anthesis, which grew from 59 to 80%, and at silking, which grew from 61 to 86%. This behavior was also observed for P3707VYH at silking, which grew from 60 to 75% when inoculated (Supplementary Figure 4F).

**FIGURE 2:**
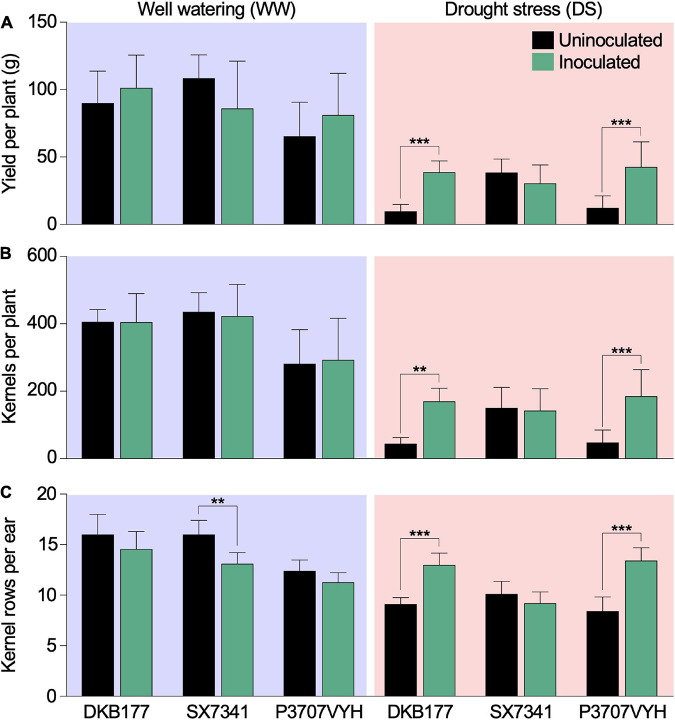
Synthetic community (SynCom) inoculation reduces the yield loss of commercial maize hybrids under DS. During DS, inoculated DKB177 and P3707VYH plants displayed higher **(A)** yield per plant (3.93× and 3.45×, respectively), **(B)** number of kernels per plant (3.87× and 3.85×, in that same order) and **(C)** number of kernel rows per ear (42 and 59%, respectively) under DS. Additional yield results are shown in Supplementary Figure 5. Values expressed as the mean ± SD. *n* ≥ 7 plants per treatment. WW, well watering; DS, drought stress; SD, standard deviation. **P ≤ 0.01 and ***P ≤ 0.001.

As expected, DS significantly reduced all yield components and aerial biomass accumulation. Differences in genotypes and in genotype × inoculum were observed for many yield components, showing specific and different responses from hybrids due to SynCom inoculation (Supplementary Table 1). SynCom inoculation significantly reduced yield loss caused by DS. Under DS, SynCom-inoculated DKB177 yielded an average of 38.7 g of seeds per plant compared to 9.8 g per plant for the uninoculated counterpart (Figure 2A). Similarly, under DS, the inoculated P3707VYH produced 42.7 g per plant compared to 12.4 g of seeds per uninoculated plant (Figure 2A). Other yield components, such as the number of kernels per plant and the number of rows per ear, were significantly higher under DS for inoculated DKB177 and P3707VYH plants than uninoculated plants. In contrast, no significant differences in yield parameters between inoculated and uninoculated plants were observed for DS-treated SX7341 (Figures 2B,C). DS-treated inoculated DKB177 and P3707VYH plants also showed 64 and 54% increases in ear diameter, respectively, compared with uninoculated plants. A tendency of increase, although not statistically significant, in ear diameter under DS was also observed for inoculated SX7341 (Supplementary Figure 5A). We also observed a significant increase of 35 and 36% in the ear length of DS-treated inoculated DKB177 and P3707VYH, respectively (Supplementary Figure 5B). Notably, among WW-treated plants, there was no significant impact of SynCom inoculation, except for SX7341, in which SynCom inoculation led to an 18% reduction in the number of kernel rows per ear (Figure 2C). Overall, there was no significant difference in the aerial biomass of mature inoculated and uninoculated plants regardless of watering regime, although aerial biomass tended to be higher in WW- and lower in DS-treated inoculated plants (Supplementary Figure 5C). The HI was higher in inoculated DKB177 and P3707VYH under both watering regimes. Particularly under DS, the HI was 4 and 3.2 times higher for inoculated DKB177 and P3707VYH, respectively, than for uninoculated plants (Supplementary Table 2).

### Daily Changes in Environmental Parameters Directly Impact Plant Physiology

**FIGURE 3:**
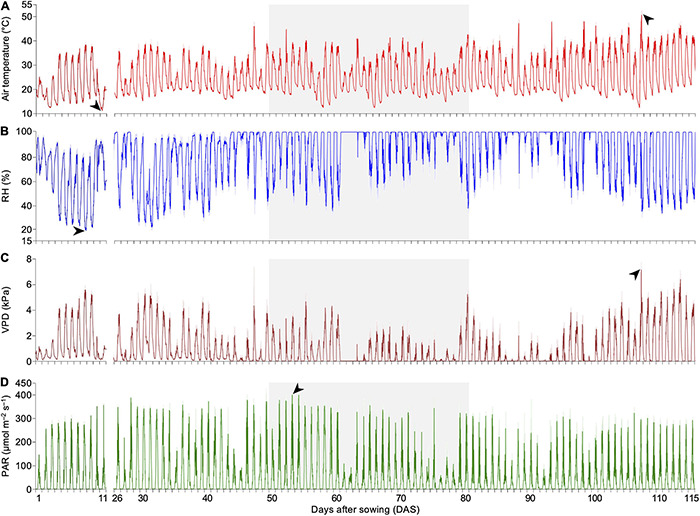
Fluctuation of environmental parameters detected by the non-invasive real-time phenotyping platform. **(A)** Air temperature, with minimum and maximum observed at 11 DAS at 5:30 am (11.35 ± 0.05°C) and 107 DAS at 2:00 pm (51.38 ± 1.93°C), respectively (black arrows). **(B)** RH, with a minimum of 18.85 ± 0.15% at 8 DAS at 4:30 pm (black arrow). **(C)** VPD, found to reach a peak of 7.17 ± 0.60 kPa at 107 DAS at 2:15 pm (black arrow), following the high air temperature variation at that moment. **(D)** PAR, with a maximum peak of 400.53 ± 1.94 μmol m^–2^ s^–1^ observed at 53 DAS at 12:00 pm (black arrow). Gray background highlights period of DS treatment. Data points were missing from 12 to 25 DAS due to an unexpected disruption of the automated measuring routine. DAS, days after sowing; RH, air relative humidity; VPD, vapor-pressure deficit; PAR, photosynthetically active radiation; DS, drought stress.

A non-invasive real-time phenotyping platform was developed to investigate parameters affecting the plant physiological response to DS when inoculated with SynCom. To ensure a complete and accurate analysis, the platform also enabled close monitoring of the environmental factors over time. During the experimental period, the average daily air temperature in the greenhouse ranged from 16.4 to 34.5°C. Particularly during the DS treatment, plants faced thermal amplitudes reaching 27.7°C and a maximum air temperature peak of 45.4°C at 52 DAS. During DS, the RH reached a minimum of 35% during the day period (6:00 am to 6:00 pm) (Figures 3A,B). Additionally, the VPD was lower during DS, with daily averages of <0.01–2.6 kPa during the day period, which intensified the DS impact (Figure 3C). Concerning light intensity, the maximum peak of PAR of 400.5 μmol m^–2^ s^–1^ was observed at 53 DAS at 12:00 pm at the beginning of DS treatment (Figure 3D).

Imaging records of plant behavior throughout the experiment revealed a constant response of plants to daily environmental variations. The air temperature, for instance, is directly correlated with the canopy structure. As air temperature increases, leaf rolling becomes proportionally more evident, especially on top leaves, which are generally most affected. At 52 DAS, for example, when plants were at the V11–V12 stage, this effect was observed from 8:00 am until 12:00 pm, when the air temperature increased from 21.4 to 36.2°C. Leaf rolling reached a maximum at 1:00 pm when the air temperature was 45.4°C (Supplementary Figures 6A–E). As the air temperature decreased in the afternoon, the leaves started to unroll (Supplementary Figures 6A,F,G). We next evaluated whether there was a correlation between air temperature, RH and PAR and the magnitude of the plant response. To this end, we monitored three sequential days (47–49 DAS) that displayed distinct air temperature amplitudes, RH and PAR. At 1:45 pm at 47 DAS, when the air temperature peaked at 45°C, RH was 64% and PAR was 321.7 μmol m^–2^ s^–1^, almost all plants tended to display more leaves rolled inward than unrolled leaves. On that day, the amplitude of air temperature reached 26°C (Supplementary Figures 7A–E). In contrast, the leaf surface area was restored for the majority of plants at 48 DAS at 2:00 pm, when the peak air temperature reached 28.6°C, with a smaller thermal amplitude of 9.6°C. At that moment, the RH was 81.1%, and the PAR was 147.9 μmol m^–2^ s^–1^ (Supplementary Figures 7A–C,F,G). An intermediate air temperature peak was observed at 12:30 pm at 49 DAS, reaching 39.4°C in a day when the amplitude of the air temperature was 21°C. Combined with a lower RH of 52.1% and reduced PAR of 105.3 μmol m^–2^ s^–1^, plants had, on average, an intermediate phenotype of leaf rolling (Supplementary Figures 7A–C,H,I).

### Synthetic Community Inoculation Decreases DKB177 Leaf Temperature During Maximum Daily Air Temperature

Given that SynCom affected plant and canopy behavior under DS, we investigated whether the inoculation could modify plant physiological responses. Plants were monitored throughout the DS period using a set of sensors to capture T_*leaf*_, sap flow, and SWC every 5 min, composing a robust data series.

**FIGURE 4:**
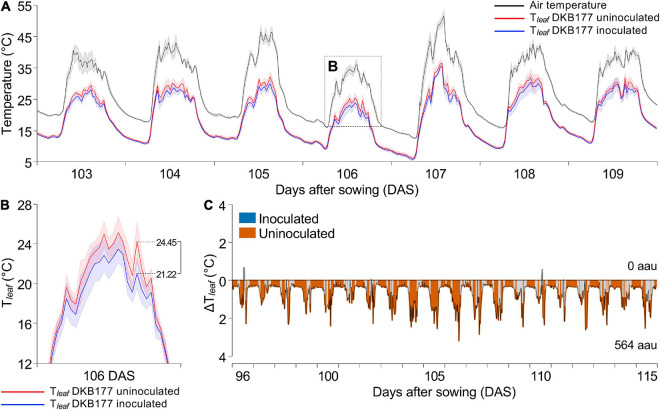
The SynCom-inoculated DKB177 plants displayed lower T_*leaf*_ than uninoculated plants. **(A)** The hybrid DKB177 showed a low T_*leaf*_ when inoculated with SynCom, especially in periods when the air temperature was high. The standard deviation is shown as the background for air temperature and both treatments. **(B)** T_*leaf*_ of uninoculated plants reaches a peak of 3.23°C higher at 106 DAS (inset from panel **A**). **(C)** The difference between T_*leaf*_ of inoculated and uninoculated plants (ΔT_*leaf*_), rounded every 30 min over time, revealed consistency in the high temperature presented by uninoculated DKB177. Values were displayed above the *x*-axis when T_*leaf*_ of inoculated plants is higher than T_*leaf*_ of uninoculated plants or below the *x*-axis when T_*leaf*_ of uninoculated plants is higher than T_*leaf*_ of inoculated plants, and colored in blue or red, respectively, when significantly different (*P* ≤ 0.05). Areas filled with light gray denote not statistically significant differences. The sums of areas in the graph above and below the *x*-axis were considered only for statistically significant differences. See Supplementary Figure 8 for the entire analyzed period. T_*leaf*_, leaf temperature; ΔT_*leaf*_, difference of T_*leaf*_; aau, arbitrary area units; DAS, days after sowing.

We first evaluated the DKB177 hybrid, which exhibited the most prominent response to SynCom inoculation. The monitoring results shows that the T_*leaf*_ of DKB177 was significantly lower in SynCom-inoculated plants under WW conditions. For example, during the 103–109 DAS period, the difference in T_*leaf*_ between uninoculated and inoculated plants (ΔT_*leaf*_) reached values of up to 3.2°C (Figures 4A,B). This difference was particularly evident in periods where VPD exceeded 2 kPa (Supplementary Figure 8A, subpanels i–iv), when high fluctuation of VPD had a strong effect on plant temperature control. During 96–115 DAS, for instance, a total of 763 (79.5%) ΔT_*leaf*_ peaks, out of 960, were found to be statistically significant. During this period, the integral area of the ΔT_*leaf*_ peaks in which uninoculated plants displayed higher T_*leaf*_ accounted for 564 arbitrary area units (aau) versus no area of inoculated plants (Figure 4C and Supplementary Figures 8A,B). We also analyzed the pattern of T_*leaf*_ of the other two hybrids (Supplementary Figures 8C,D). During the entire experiment, WW-treated inoculated P3707VYH also displayed lower T_*leaf*_ than uninoculated plants, on average (68 against 195 aau, respectively) (Supplementary Figure 8D). Curiously, although the average T_*leaf*_ of inoculated SX7341, in general, tended to be higher (313 against 234 aau for inoculated and uninoculated, respectively), during days when VPD was >2 kPa, uninoculated plants displayed higher T_*leaf*_, with peaks of ΔT_*leaf*_ summing to 16, 15, 27, and 171 against 30, 2, 4, and 39 aau for SynCom-inoculated plants (Supplementary Figures 8A,C).

An interesting response to SynCom was found for the SX7341 plants. In contrast to that observed for WW-treated DKB177, in which T_*leaf*_ differences between inoculated and uninoculated plants were observed throughout the whole day, major differences in T_*leaf*_ for WW-treated inoculated SX7341 plants were observed at the air temperature peaks, suggesting a delayed T_*leaf*_ increase phenomenon (Supplementary Figures 9A,B). For example, from 95 to 114 DAS, at the air temperature peaks, the T_*leaf*_ of uninoculated plants was, in general, higher than the T_*leaf*_ of the inoculated plants. The integrated areas of T_*leaf*_ differences (ΔT_*leaf*_) that were statistically significant summed to 164 and 46 aau for uninoculated and inoculated plants, respectively (Supplementary Figure 9B). However, inspections into subperiods revealed that the majority of peaks when the T_*leaf*_ of uninoculated plants were higher than the T_*leaf*_ of inoculated plants occurred from 9:00 am to 12:00 pm (Supplementary Figure 9C), while late peaks when the T_*leaf*_ of inoculated plants was higher than the T_*leaf*_ of uninoculated plants were mostly observed from 12:00 pm to 3:00 pm (Supplementary Figure 9D).

At the late stages of DS treatment (22–29 days of DS; 72–79 DAS), the integrated ΔT_*leaf*_ areas among inoculated and uninoculated plants revealed that SynCom increased the T_*leaf*_ of all hybrids. During an 8-day period immediately before rehydration, the statistically significant differences in T_*leaf*_ summed to 69 against 3 aau for inoculated and uninoculated DKB177 (Supplementary Figure 10A), 53 aau against no area for SX7341 (Supplementary Figure 10B) and 48 against 4 aau for P3707VYH (Supplementary Figure 10C). The analysis of SWC revealed that plants presented significant differences in soil moisture when comparing WW and DS conditions for each hybrid (Supplementary Figures 10D–F).

### Synthetic Community Differentially Modulates Sap Flow at Well Watering and Drought Stress Conditions

**FIGURE 5:**
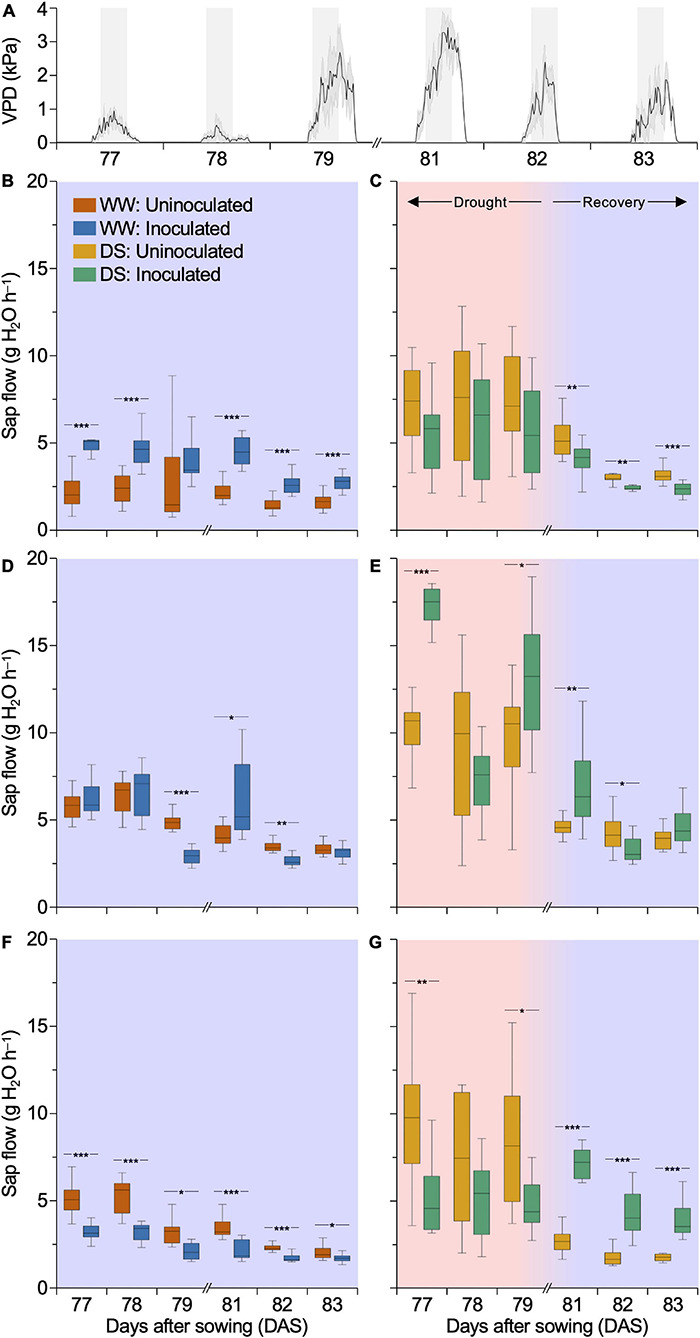
Synthetic community (SynCom) inoculation affects the sap flow of maize hybrids. **(A)** Fluctuation of VPD (kPa) from 77 to 83 DAS. The gray background highlights daily windows from 10:00 am to 4:00 pm, periods considered to measure sap flow of DKB177 **(B,C)**, SX7341 **(D,E)**, and P3707VYH **(F,G)**. Box plots are shown for WW- **(B,D,F)** and DS-treated/rehydrated **(C,E,G)** plants. Rehydration was performed at 80 DAS. **(B)** In WW, SynCom leads to an increase in DKB177 sap flow by up to 2.22×. **(C)** Inoculated DKB177 plants tended to have their sap flow reduced in late stages of DS (77–79 DAS). During recovery (81–83 DAS), this reduction significantly reached up to 25.9%. **(D)** SX7341 presented an undefined pattern of sap flow under WW the regime. **(E)** A lack of pattern was also found during the DS and rehydration periods. **(F)** WW-treated P3707VYH had its sap flow reduced by up to 39.7% when inoculated, the same effect found in DS **(G)**. **(G)** During recovery, SynCom inoculation led to a shift in sap flow of P3707VYH with an increase of up to 2.57× compared to the control. VPD: vapor-pressure deficit; WW, well watering; DS, drought stress; DAS, days after sowing. *P ≤ 0.05, **P ≤ 0.01, and ***P ≤ 0.001.

The effect of SynCom inoculation on T_*leaf*_ led us to hypothesize whether inoculation treatment influenced plant water usage. We monitored the sap flow of plants 3 days before (late DS stages) and 3 days after rehydration initiation, periods in which variations in water usage should be mostly contrasting between treatments. The analysis of sap flow considered a period between 10:00 am and 4:00 pm, when plant transpiration was maximal due to the high VPD (Figure 5A). Under WW, inoculated DKB177 showed a sap flow 1.7–2.2 times higher than that of the uninoculated plants, consistent with the lower T_*leaf*_ observed for these plants. The most pronounced difference was found at 77 DAS when the sap flow was 2.2 and 5 g H_2_O h^–1^ for uninoculated and inoculated plants, respectively (Figure 5B). On the other hand, under DS, inoculated DKB177 displayed reduced sap flow compared to the uninoculated plants, which might be related to the fact that these plants did not loss turgor and did not bend under SDS (Figure 1C and Supplementary Figures 3F,G). In the first 3 days after rehydration (81–83 DAS), the sap flow of inoculated DKB177 was 20.7–25.9% lower (2.3–4 g H_2_O h^–1^) than that of uninoculated plants (3.1–5.3 g H_2_O h^–1^) (Figure 5C). During the 6-day window of DS/rehydration, SX7341 presented an undefined pattern of sap flow for inoculated and uninoculated plants under WW conditions (Figure 5D). The same lack of pattern was observed under DS and rehydration. At late stages of DS (79 DAS), inoculated SX7341 plants had a significant increase in sap flow compared to uninoculated plants (13 versus 9.6 g H_2_O h^–1^, respectively), a pattern that was also observed on the day immediately after rehydration (81 DAS), with 6.9 versus 4.6 g H_2_O h^–1^ sap flow for inoculated and uninoculated plants, respectively (Figure 5E). WW-treated inoculated P3707VYH consistently decreased the sap flow by 15–39.7% compared to uninoculated plants. The most pronounced effect of SynCom inoculation was found at 78 DAS, with a sap flow of 3.2 against 5.3 g H_2_O h^–1^ for inoculated and uninoculated plants, respectively (Figure 5F). Remarkably, a clear shift was found during the 3 days following rehydration (81–83 DAS). The inoculated P3707VYH plants increased sap flow by 2.2–2.6 times compared to the uninoculated plants. On average, the sap flow of the uninoculated plants was 1.8–2.8 g H_2_O h^–1^ compared to 4–7.1 g H_2_O h^–1^ of the inoculated plants (Figure 5G), which is consistent with the observation that inoculated P3707VYH presented a faster recovery than the uninoculated plants (Figure 1D and Supplementary Figure 3H).

### Members of Synthetic Community Robustly Colonize Maize Plants and Reshape Resident Microbiota

**FIGURE 6:**
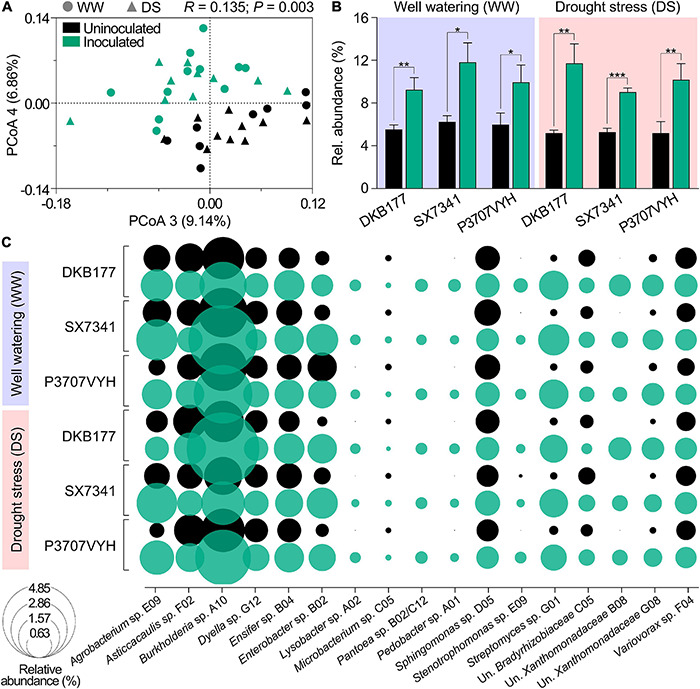
Members of SynCom robustly colonize different maize hybrids. **(A)** PCoA of the Bray–Curtis dissimilarity matrix of inoculated and uninoculated plants. **(B)** Relative abundance of OTUs present in SynCom in WW- and DS-treated inoculated and uninoculated DKB177, SX7341, and P3707VYH hybrids. **(C)** Relative abundance of community-based isolates in SynCom in WW- and DS-treated inoculated and uninoculated maize hybrids. OTUs of community-based isolates identified as robust colonizers are individually highlighted in Supplementary Table 3. Values expressed as the mean ± SD. PCoA, principal coordinates analysis; SD, standard deviation; Un., unknown. *P ≤ 0.05, **P ≤ 0.01, and ***P ≤ 0.001.

We also asked if the impact of inoculation on plant physiology were correlated with colonization by SynCom members. To address this question, we profiled the root microbiome of inoculated and uninoculated plants through 16S rRNA amplicon sequencing. Differences in the bacterial community structure assemblages were first analyzed using principal coordinates analysis (PCoA) of the Bray–Curtis dissimilarity matrix. Inoculated and uninoculated plants clustered separately (ANOSIM; *R* = 0.135; *P* = 0.003), indicating that inoculation modified microbial community assemblage in plant roots. No significant difference was observed in community composition between WW- and DS-treated plants (Figure 6A). OTUs representative of SynCom members accounted for 9.3, 11.8, and 9.9% of inoculated DKB177, SX7341, and P3707VYH under WW and 11.7, 9, and 9.6% under DS, respectively (Figure 6B). SynCom-associated OTUs were then individually evaluated for their relative abundance. In total, 23 OTUs were assigned to 17 SynCom community-based isolates: *Agrobacterium* sp. E09, *Asticcacaulis* sp. F02, *Burkholderia* sp. A10, *Dyella* sp. G12, *Ensifer* sp. B04, *Enterobacter* sp. B02, *Lysobacter* sp. A02, *Microbacterium* sp. C05, *Pantoea* sp. B02/C12, *Pedobacter* sp. A01, *Sphingomonas* sp. D05, *Stenotrophomonas* sp. E09, *Streptomyces* sp. G01, unknown Bradyrhizobiaceae C05, unknown Xanthomonadaceae B08, unknown Xanthomonadaceae G08, and *Variovorax* sp. F04 (Figure 6C). From the assigned OTUs, 14 OTUs were considered robust colonizers for at least one hybrid at WW, while 16 OTUs were found in the same category under DS. Among these, only two, *Agrobacterium* sp. E09 and *Enterobacter* sp. B02, were exclusively found as robust colonizers in at least one hybrid in DS-treated plants but not in plants under WW. Overall, OTUs were classified as non-robust colonizers when considering all hybrids and treatments. Among robust colonizer groups, *Ensifer* sp. B04 was the most efficient colonizer, comprising 0.78−0.86% and 0.72−0.87% of the relative abundance among the three hybrids in WW- and DS-treated plants, respectively. *Variovorax* sp. F04 and *Streptomyces* sp. G01 colonized all hybrids at 0.41–0.49% and 0.37−0.49% (under WW) and 0.42−0.46% and 0.31−0.51% (under DS), respectively (Figure 6C and Supplementary Table 3). Among 17,437 identified OTUs belonging to resident plant microbiota (apart from those belonging to SynCom), 632 displayed a significant shift in inoculated compared to uninoculated plants for both WW- and DS-treated plants, indicating that members of resident microbiota were enriched or depleted in SynCom-inoculated plants (Supplementary Figure 11). Overall, these results indicate that SynCom members efficiently colonize plant root systems, displaced naturally occurring microbes and recruit groups that may help boost the inoculation impact.

## Discussion

### A Synthetic Community That Mitigates Drought Stress in Maize

Under water restriction, decreased photosynthesis, stomatal conductance and CO_2_ assimilation result in reduced yield (Chaves et al., 2003). To cope with DS, plants activate several protective mechanisms, including associations with beneficial microorganisms that help plants survive unfavorable circumstances. However, exploring the benefits and functions of the plant microbiota is still technically challenging given the diversity and complexity of microbial communities. A promising approach is the use of SynComs, which are consortia of microorganisms that mimic, to some extent, the observed structure of the plant microbiome under natural conditions (Vorholt et al., 2017). Multiple SynComs have been recently investigated for playing important roles in terms of mitigation of different plant stresses, such as protection against pathogens in maize (Niu et al., 2017), more efficient nitrogen fixation in rice (Zhang et al., 2019) and enhanced phosphate starvation responses in *Arabidopsis* (Castrillo et al., 2017). In this work, we evaluated the influence of a SynCom composed of microbes from the sugarcane core microbiome (de Souza et al., 2016, 2019; Armanhi et al., 2018) in three commercial maize hybrids grown to maturity under drought.

Abiotic stresses are known to affect an array of physiological and molecular processes, including stomatal closure, in an attempt to prevent turgor loss (Mahajan and Tuteja, 2005). In this work, leaf rolling, a sensitive indicator of water restriction in maize (Song et al., 2018), was the most premature visual response of all plants subjected to drought. Under SDS, the uninoculated plants of the three maize hybrids were severely affected, resulting in plant bending, a well-known response indicating cell turgor loss. In contrast, SynCom-inoculated maize hybrids retained turgor, either partially or permanently. In DKB177, for instance, SynCom inoculation induced physiological mechanisms that prevented plant bending even in late stages of SDS. Meanwhile, a faster recovery after rehydration, a critical process associated with drought tolerance in maize, was observed for P3707VYH.

The benefit of microbe-inoculated plants subjected to DS has been reported for plant biomass and yield (Rubin et al., 2017). Here, we show that SynCom inoculation reduced the yield loss of maize hybrids under DS, indicating that these microbes play a significant role when plant homeostasis is perturbed, as in regular conditions plants expressed their maximum genetic potential. Under stressful conditions, plants are known to recruit sets of microbes with abilities to mitigate specific detrimental effects (Naylor et al., 2017; Beirinckx et al., 2020). We observed a higher yield of inoculated DKB177 and P3707VYH hybrids under DS, with a tendency toward reduction in aerial biomass and increased HI. This may be due to the remobilization of assimilates from straw to the grains through specific hormonal regulation and enzymatic activities when plants face DS in reproductive stages, as observed for other crops (Plaut et al., 2004), which is supported by increased HI in inoculated plants. Although SynCom inoculation did not mitigate DS impact in terms of flowering time, it reduced the number of plants lacking pollen shed for DKB177 and silk extrusion for both DKB177 and P3707VYH, which is consistent with increased yield in inoculated plants. The higher yield of inoculated plants may indicate carbon mobilization after fertilization by reducing carbon limitation to early kernel development (Oury et al., 2016).

Adverse environmental conditions, plant–microbe inter- actions and the plant genetic background can collectively contribute to seedling emergence. Upon inoculation, we observed reduced seedling emergence for DKB177 and more uneven seedling emergence for both DKB177 and P3707VYH. Although uneven seedling emergence negatively impacts grain yield and the HI of maize (Liu et al., 2004), we observed no direct correlation with plant yield. The reduced number of seedlings may be explained by the high density of inoculated bacteria at the beginning of seed germination, given that seedlings are a vulnerable stage in plant development (Nelson, 2018). Additionally, at early germination stages, the high density of microbes may compete for the available nutrients in the soil (Kozdrój et al., 2004; Zakeel and Safeena, 2019). Since hybrids were differentially affected by SynCom inoculation, it is more likely that plant genetic background and plant-SynCom crosstalk might have been involved.

### Synthetic Community Differentially Impacts Maize Hybrids by Modulating Leaf Temperature Control and Water Usage Optimization

An existing hurdle to plant phenotyping relies on the lack of appropriate instruments capable of non-invasive and real-time plant behavior assessments. Destructive methods for plant phenotyping are often used but may not be operationally desirable, as they require an exponential number of samples. In addition to manual instruments conventionally employed, most platforms for plant phenotyping are mainly based on either sensor-to-plant (De Diego et al., 2017) or plant-to-sensor setups (Fahlgren et al., 2015), in which automated systems harboring cameras/sensors or plants routinely move along a platform. However, as plant growth is a dynamic process, the majority of phenotyping platforms may be restricted to a limited number of data points and specific timepoints (Halperin et al., 2017; Rouphael et al., 2018). In this work, the real-time monitoring of individual plants allowed the establishment of a one-to-one (plant–sensor) setup and assured that even small variations in plant physiology along a single day period were detected. Over time, the environmental and physiological high-resolution data allowed us to record instant plant responses to small daily environmental perturbations in detail.

The results presented in this work revealed that SynCom inoculation directly influenced T_*leaf*_ under environmental conditions fluctuations. Under DS, the reduction in transpiration typically induces higher canopy temperatures as a consequence of changes in stomatal conductance. The variability in T_*leaf*_ has been traditionally applied as an indicator of DS symptoms (González-Dugo et al., 2006). We surprisingly observed that SynCom-inoculated plants of the DKB177 hybrid displayed decreased T_*leaf*_ under WW conditions. This physiological behavior would be advantageous for plants, especially because elevated temperatures cause a reduction in enzyme activities, such as RuBisCO, and impact plant growth (Crafts-Brandner and Salvucci, 2002). We also observed a temporally segmented response of T_*leaf*_ for the SynCom-inoculated SX7341 hybrid that showed better T_*leaf*_ control at the beginning of the daily air temperature increase, suggesting that SynCom may contribute to plant primary mechanisms associated with a given environmental disturbance. To our knowledge, this is the first study to describe such plant physiological attributes induced by microbes.

Given that plant transpiration is directly affected by stomatal aperture, sap flow is often used to monitor plant water status. Particularly under DS, xylem sap transports signaling molecules from roots to shoots that signal to reduce plant growth and transpiration (Alvarez et al., 2008). The observation that the sap flow of inoculated DKB177 under WW was significantly higher than that in uninoculated plants correlates with the observed reduced T_*leaf*_. However, under DS, the opposite effect is shown, as SynCom inoculation did not reduce T_*leaf*_, which is correlated with a significant reduction in sap flow, particularly for DKB177 and P3707VYH plants. Interestingly, the three different maize hybrids presented distinct physiological behavior during recovery from DS, whether maintaining reduced sap flow in stalks (DKB177) or displaying a significant shift in sap flow and recovery capability upon rehydration (P3707VYH). Despite not being evaluated, increased root branching is expected for SynCom-inoculated plants since more developed root systems as a whole were observed for inoculated early stage maize plants (Armanhi et al., 2018). The optimization of water usage and cell turgor maintenance might be associated with osmolyte production induced by SynCom inoculation, a function that should be highly dependent on the genetic background of the three distinct maize hybrids.

In the present study, no significant differences were found between WW and DS concerning the colonization robustness of SynCom members of inoculated plants. Nevertheless, we found that the majority of SynCom members robustly colonized the roots of inoculated plants, consistent with our previous results (Armanhi et al., 2018; de Souza et al., 2019). The SynCom members *Agrobacterium* sp. E09, *Burkholderia* sp. A10, *Ensifer* sp. B04, *Lysobacter* sp. A02, *Pedobacter* sp. A01, *Stenotrophomonas* sp. E09, *Streptomyces* sp. G01 and *Variovorax* sp. F04 were most prevalent and may have been responsible for the beneficial impact on maize hybrids under DS. Curiously, the SynCom members *Enterobacter* sp. B02, *Pantoea* sp. B02/C12, unknown Xanthomonadaceae B08 and unknown Xanthomonadaceae G08, previously found to be non-robust colonizers in early stage maize plants (Armanhi et al., 2018; de Souza et al., 2019), displayed robust colonization in mature plants, suggesting a functional role at the late stages of plant development.

Several bacterial traits associated with DS tolerance in plants have been proposed. For example, the production of exopolysaccharides (EPS) by EPS-producing bacteria was shown to increase the leaf relative water content of maize, thus improving plant fitness under DS (Naseem and Bano, 2014). Bacterial groups present in the SynCom are enriched in genes related to EPS production (de Souza et al., 2019). Many other SynCom-encoded traits related to plant water usage, such as the production of osmolytes that sustain higher tissue water potential, may also contribute to plant DS tolerance. Plant osmolytes are known to increase water influx in cells, thus playing roles in cell turgor maintenance (Bartels and Sunkar, 2005). We hypothesize that members of the SynCom may also stimulate plant osmolyte production for their own benefit, as they are enriched in ABC-type transporters for proline, glycine betaine, sugars, and amino acids, among other compounds (de Souza et al., 2019).

The microbial root profiling of the three hybrids revealed a shift in the soil/seed-borne microbial composition of SynCom-inoculated plants, suggesting reshaping of the natural microbiota. Although the view of a functional plant–microbiome bond is more parsimonious regardless of microbial taxonomic affiliation, taxonomy still may provide preliminary clues for the role of beneficial microbes in plant development and response to environmental perturbations. Microbial groups in the Proteobacteria phylum, for instance, were enriched in all inoculated plants of the three hybrids, particularly families already described as harboring plant beneficial microbes, such as Bradyrhizobiaceae (Shaharoona et al., 2006), Burkholderiaceae (Stoyanova et al., 2007), Caulobacteraceae (Pepe et al., 2013), Comamonadaceae (Belimov et al., 2005), and Phyllobacteriaceae (Wani et al., 2007). Thus, our findings suggest that SynCom inoculation recruits and enriches such beneficial microbial groups naturally found in low abundance in the plant/soil microbiota. This recruitment may have helped to sustain plant homeostasis under stressful DS conditions.

## Conclusion

The beneficial impact of SynCom inoculation on the plant physiological parameters of commercial maize hybrids subjected to DS was monitored in real time. Detailed information on physiological behavior during the developmental process and in response to DS was made possible by continuous monitoring using a non-invasive phenotyping platform comprised of sensors at the individual plant level and high-quality imaging. Inoculation with SynCom increased drought tolerance and reduced yield loss. The collected dataset provided significant evidence that SynCom inoculation modulates plant T_*leaf*_ and optimizes water usage. Colonization profiling revealed that members of SynCom robustly colonized plant roots and recruited beneficial soil/seed-borne bacterial members, thus increasing plant resilience to DS. Our findings suggest that although the molecular mechanisms of plant–SynCom interactions remain to be elucidated, a better comprehension of crop functional trait responses to drought can provide evidence of their potential to enhance plant performance.

## Data Availability Statement

Sequencing raw data were deposited in the Sequence Read Archive (SRA) database under the accession number PRJNA384812. All physiological and environmental data generated and analyzed during this study can be found in Supplementary Datasets 1, 2.

## Author Contributions

JA and RdS designed the SynCom and the inoculation experiment, prepared sequencing libraries of the 16S rRNA gene for microbe identification, and performed bioinformatics analyses. JA, RdS, and BB prepared the SynCom and performed the inoculation experiment, plant phenotyping and sampling for microbial profiling. JA designed, built, installed, and analyzed the data from the plant phenotyping platform with significant inputs of RdS, JY, and PA. JY performed statistical analysis of yield components. JA wrote the manuscript with contributions from all authors. RdS and PA critically revised the manuscript. All authors read and approved the final manuscript.

## Conflict of Interest

The authors declare that the research was conducted in the absence of any commercial or financial relationships that could be construed as a potential conflict of interest.

## Publisher’s Note

All claims expressed in this article are solely those of the authors and do not necessarily represent those of their affiliated organizations, or those of the publisher, the editors and the reviewers. Any product that may be evaluated in this article, or claim that may be made by its manufacturer, is not guaranteed or endorsed by the publisher.

## Footnotes

1
http://www.ffmpeg.org

2
www.graphpad.com

## Supplementary Material

Supplementary Figure 1 Experimental design to evaluate the impact of SynCom in three different commercial hybrids of maize under DS.

Supplementary Figure 2 Sensors and materials used for the non-invasive real-time phenotyping platform.

Supplementary Figure 3 Differential phenotypic responses of three commercial hybrids of maize (DKB177, SX7341, and P3707VYH) to the presence of SynCom under DS.

Supplementary Figure 4 SynCom inoculation differentially affects seedling emergence timing and reproductive stages of maize.

Supplementary Figure 5 SynCom differentially impacts yield-related parameters and plant biomass of commercial hybrids of maize under DS conditions.

Supplementary Figure 6 Maize canopy structure throughout a day as a function of air temperature detected by the non-invasive real-time phenotyping platform.

Supplementary Figure 7 Continuous plant monitoring revealed a direct influence of natural environmental variables in canopy aspect.

Supplementary Figure 8 SynCom differentially affects maize hybrids by dramatically changing T_*leaf*_ control under WW conditions.

Supplementary Figure 9 SynCom delays the T_*leaf*_ increase in SX7341 maize plants under WW conditions.

Supplementary Figure 10 SynCom induces an increase in the T_*leaf*_ of maize plants under DS conditions.

Supplementary Figure 11 Enriched and depleted resident microbial groups in inoculated plants.

Supplementary Table 1 Three-way ANOVA for phenotypic parameters (yield per plant, kernels per plant, kernel rows per ear, ear diameter, ear length, and aerial biomass) of genotype, irrigation regime, inoculation and all their possible interactions.

Supplementary Table 2 Harvest index (HI) for inoculated and uninoculated DKB177, SX7341, and P3707VYH under WW and DS conditions.

Supplementary Table 3 Relative abundance of community-based isolates considered robust colonizers in WW- and DS-treated inoculated DKB177, SX7341, and P3707VYH hybrids.

Supplementary Movie 1 Time-lapse movie of late stages of severe drought and rehydration of DKB177, SX7341, and P3707VYH maize hybrids inoculated or uninoculated with SynCom.

Supplementary Dataset 1 Physiological data collected by the plant phenotyping platform.

Supplementary Dataset 2 Environmental data collected by the plant phenotyping platform.
