# Supplementary material for: Modulating Drought Stress Response of Maize by a Synthetic Bacterial Community
Source: Front Microbiol. 2021 Oct 21;12:747541. doi: 10.3389/fmicb.2021.747541 (PMC8566980; doi:10.3389/fmicb.2021.747541)
Supplement: Supplementary Figure 1 — Experimental design to evaluate the impact of SynCom in three different commercial hybrids of maize under DS. [file Image_1.pdf]

doi: 10.3389/fmicb.2021.747541

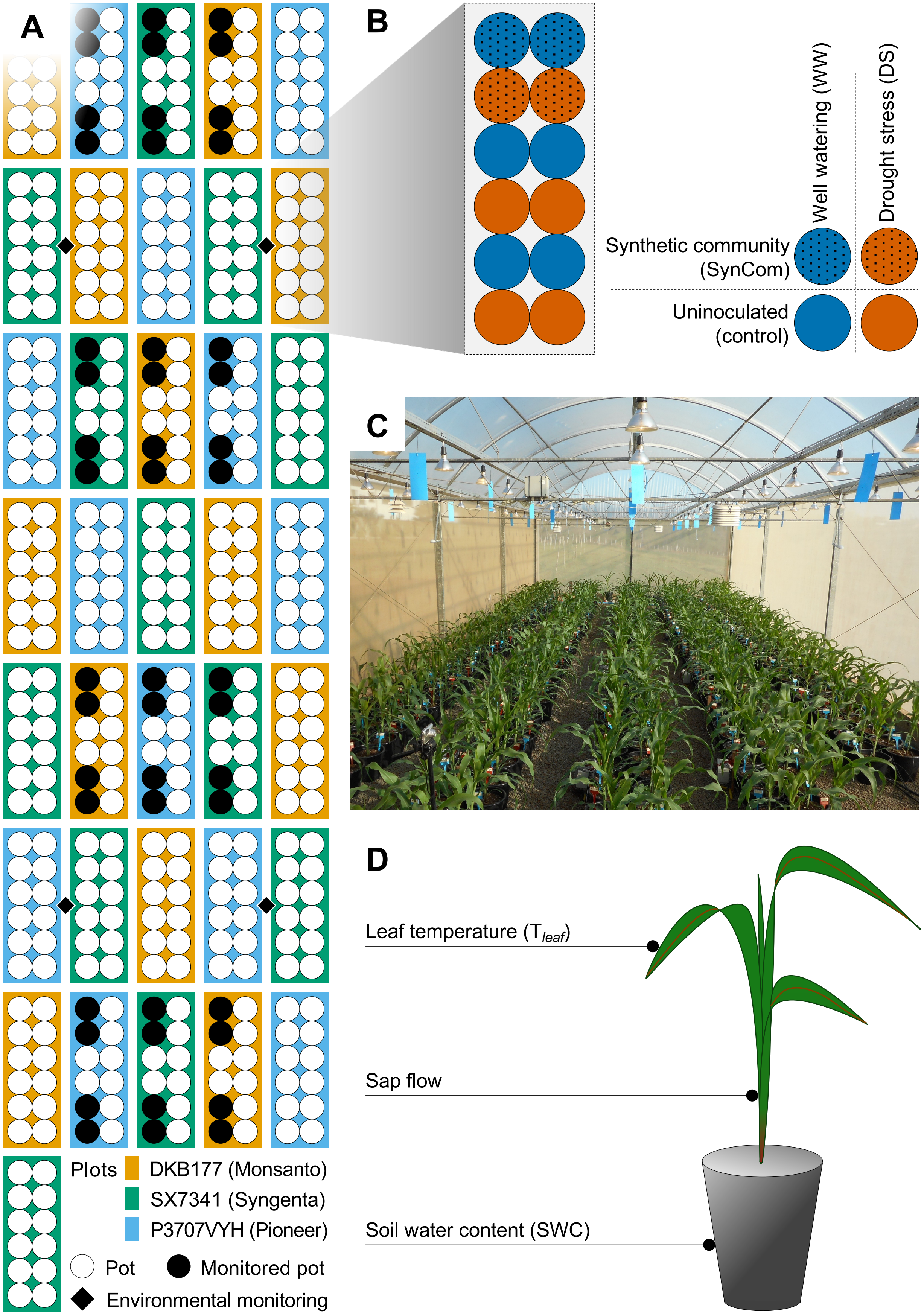

SUPPLEMENTARY FIGURE 1 | Experimental design to evaluate the impact of SynCom in three different commercial hybrids of maize under DS. **(A)** Experimental layout showing maize plants of hybrids DKB177, SX7341, and P3707VYH placed in 36 plots of 12 plants each, with 48 plants fully equipped with a set of sensors for continuous monitoring. Environmental data were obtained from four stations located along the greenhouse. **(B)** Plots were distributed in a twin row experimental design in a 90-m² greenhouse **(C)**. **(D)** Monitored plants (shown as black circles in panel **A**) were fully equipped with sensors for T_{leaf}, sap flow and SWC. DS, drought stress; T_{leaf}, leaf temperature; SWC, soil water content.
