# Supplementary material for: Modulating Drought Stress Response of Maize by a Synthetic Bacterial Community
Source: Front Microbiol. 2021 Oct 21;12:747541. doi: 10.3389/fmicb.2021.747541 (PMC8566980; doi:10.3389/fmicb.2021.747541)
Supplement: Supplementary Figure 2 — Sensors and materials used for the non-invasive real-time phenotyping platform. [file Image_2.pdf]

doi: 10.3389/fmicb.2021.747541

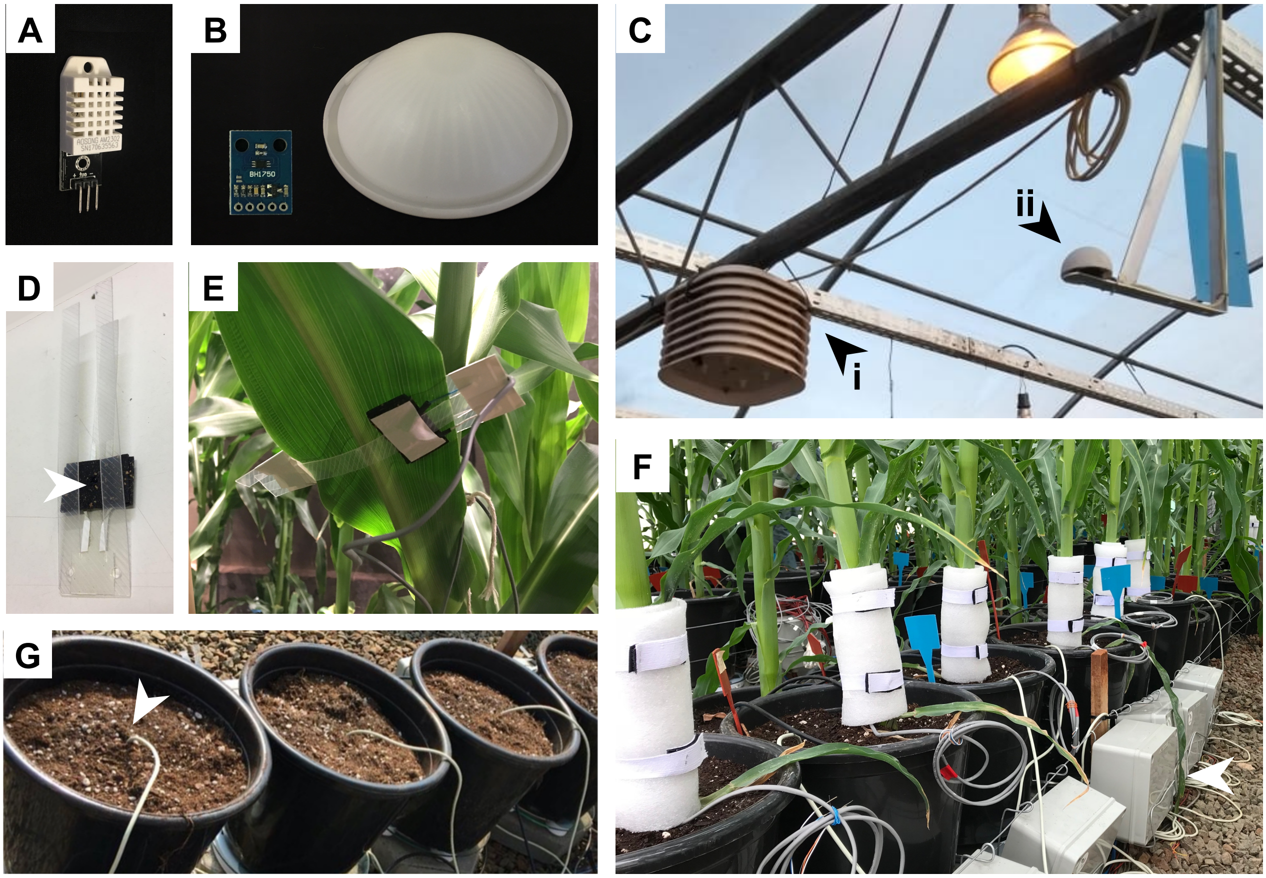

SUPPLEMENTARY FIGURE 2 | Sensors and materials used for the non-invasive real-time phenotyping platform. **(A)** Sensor for air temperature and RH. **(B)** Module for measurement of PAR (left) and light diffuser bulb (right) used. **(C)** Bottom side view of the environmental sensors positioned at the top of the greenhouse with the air temperature and RH sensor protected by a solar radiation shield (subpanel *i*) and the module for the PAR sensor inside the light diffuser bulb (subpanel *ii*). **(D)** Sensor for T_{leaf} constructed with a malleable polypropylene sheet and cork, with a thermistor placed inside the 1-mm hole (white arrow). **(E)** T_{leaf} sensor positioned at the abaxial leaf surface. **(F)** Apparatus for the measurement of sap flow in plant stems and boxes of data control and acquisition for each pot (white arrow). **(G)** Capacitive soil moisture sensors positioned in the pots (white arrow). RH, air relative humidity; PAR, photosynthetically active radiation; T_{leaf}, leaf temperature.
