# Supplementary material for: Modulating Drought Stress Response of Maize by a Synthetic Bacterial Community
Source: Front Microbiol. 2021 Oct 21;12:747541. doi: 10.3389/fmicb.2021.747541 (PMC8566980; doi:10.3389/fmicb.2021.747541)
Supplement: Supplementary Figure 3 — Differential phenotypic responses of three commercial hybrids of maize (DKB177, SX7341, and P3707VYH) to the presence of SynCom under DS. [file Image_3.pdf]

doi: 10.3389/fmicb.2021.747541

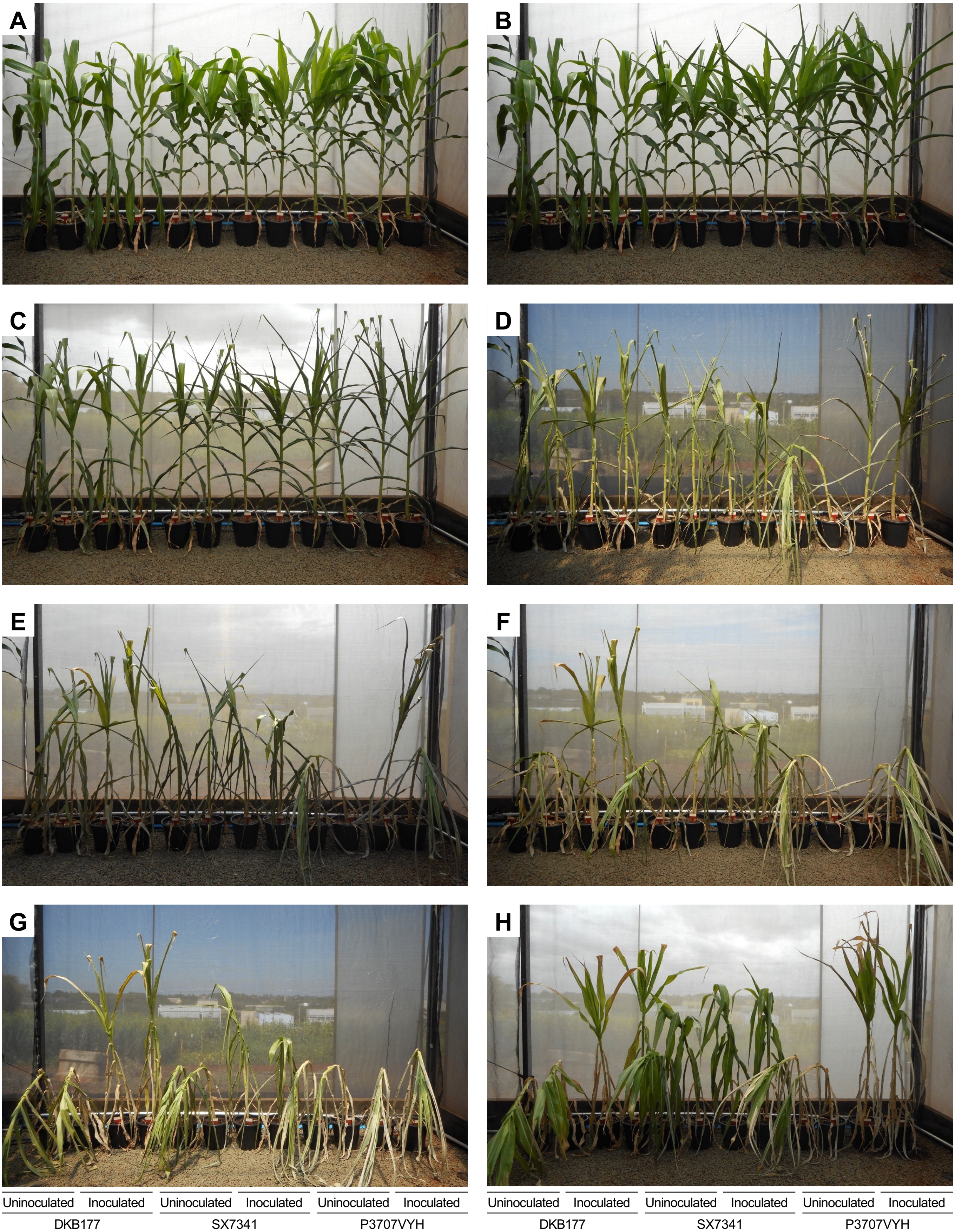

SUPPLEMENTARY FIGURE 3 | Differential phenotypic responses of three commercial hybrids of maize (DKB177, SX7341, and P3707VYH) to the presence of SynCom under DS. **(A)** After 26 days of SDS (76 DAS, at 10:00 am), all plants had their leaves briefly unrolled for the last time in the morning. **(B)** Both SX7341 and P3707VYH presented their leaves permanently rolled inward at 3:00 pm after 26 days of SDS (76 DAS). **(C)** At 28 days after SDS (78 DAS, at 3:00 pm), all hybrids exhibited a premature fall of older leaves, regardless of inoculation or not. **(D)** Three days later, uninoculated P3707VYH was completely bent after 31 days of SDS (81 DAS, at 10:00 am) in contrast with inoculated plants. **(E)** After 32 days of SDS (82 DAS, at 3:00 pm), uninoculated DKB177 and SX7341 started to bend and were completely bent at 83 DAS at 10:00 am **(F)**, in contrast to their inoculated treatments. At that stage **(F)**, inoculated SX7341 remained partially bent, while inoculated DKB177 remained completely straight. **(G)** All uninoculated hybrids were completely bent over the ground after 34 days of SDS (84 DAS, at 11:00 am), as were both inoculated P3707VYH (completely bent) and SX7341 (partially bent). The most pronounced response was found for P3707VYH, in which leaves were completely shriveled, dry and brown. **(H)** Two days after rehydration (86 DAS, at 3:00 pm), inoculated SX7341 and P3707VYH completely straightened in opposition to the uninoculated plants. Inoculated DKB177, which always remained straight, also had greener leaves that opened after rehydration. DS, drought stress; SDS, severe drought stress; DAS, days after sowing.
