# Supplementary material for: Modulating Drought Stress Response of Maize by a Synthetic Bacterial Community
Source: Front Microbiol. 2021 Oct 21;12:747541. doi: 10.3389/fmicb.2021.747541 (PMC8566980; doi:10.3389/fmicb.2021.747541)
Supplement: Supplementary Figure 4 — SynCom inoculation differentially affects seedling emergence timing and reproductive stages of maize. [file Image_4.pdf]

doi: 10.3389/fmicb.2021.747541

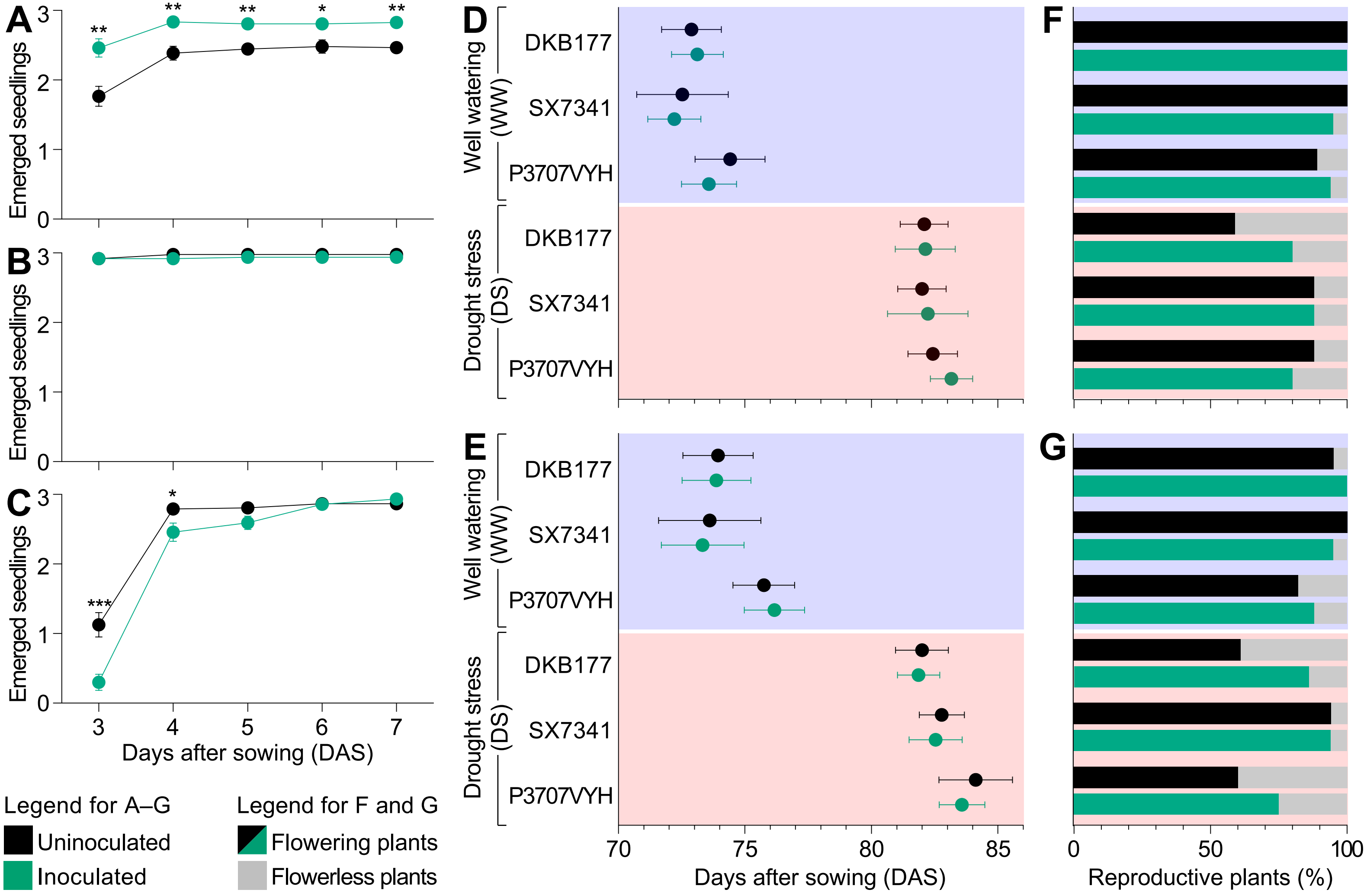

SUPPLEMENTARY FIGURE 4 | SynCom inoculation differentially affects seedling emergence timing and reproductive stages of maize. **(A–C)** Daily counting of emerged seedlings for DKB177 **(A)**, SX7341 **(B)**, and P3707VYH **(C)** revealed that hybrids differentially responded to SynCom inoculation. **(D,E)** The onset of reproductive stages, anthesis **(D)** and silking **(E)**, were remarkably delayed in DS, showing no significant impact of SynCom inoculation. **(F,G)** Percentage of flowering and flowerless plants at anthesis **(F)** and silking **(G)** for inoculated and uninoculated DKB177, SX7341, and P3707VYH under the WW and DS regimes. The lack of flowers increased under DS for DKB177 (anthesis and silking) as well as for P3707VYH (silking). Values expressed as the mean ± SEM **(A–C)** and mean ± SD **(D,E)**. $n \geq 7$ plants per treatment. WW, well watering; DS, drought stress; DAS, days after sowing; SEM, standard error of the mean; SD, standard deviation. $^{*}P \leq 0.05$, $^{**}P \leq 0.01$, and $^{***}P \leq 0.001$.
