# Supplementary material for: Modulating Drought Stress Response of Maize by a Synthetic Bacterial Community
Source: Front Microbiol. 2021 Oct 21;12:747541. doi: 10.3389/fmicb.2021.747541 (PMC8566980; doi:10.3389/fmicb.2021.747541)
Supplement: Supplementary Figure 5 — SynCom differentially impacts yield-related parameters and plant biomass of commercial hybrids of maize under DS conditions. [file Image_5.pdf]

doi: 10.3389/fmicb.2021.747541

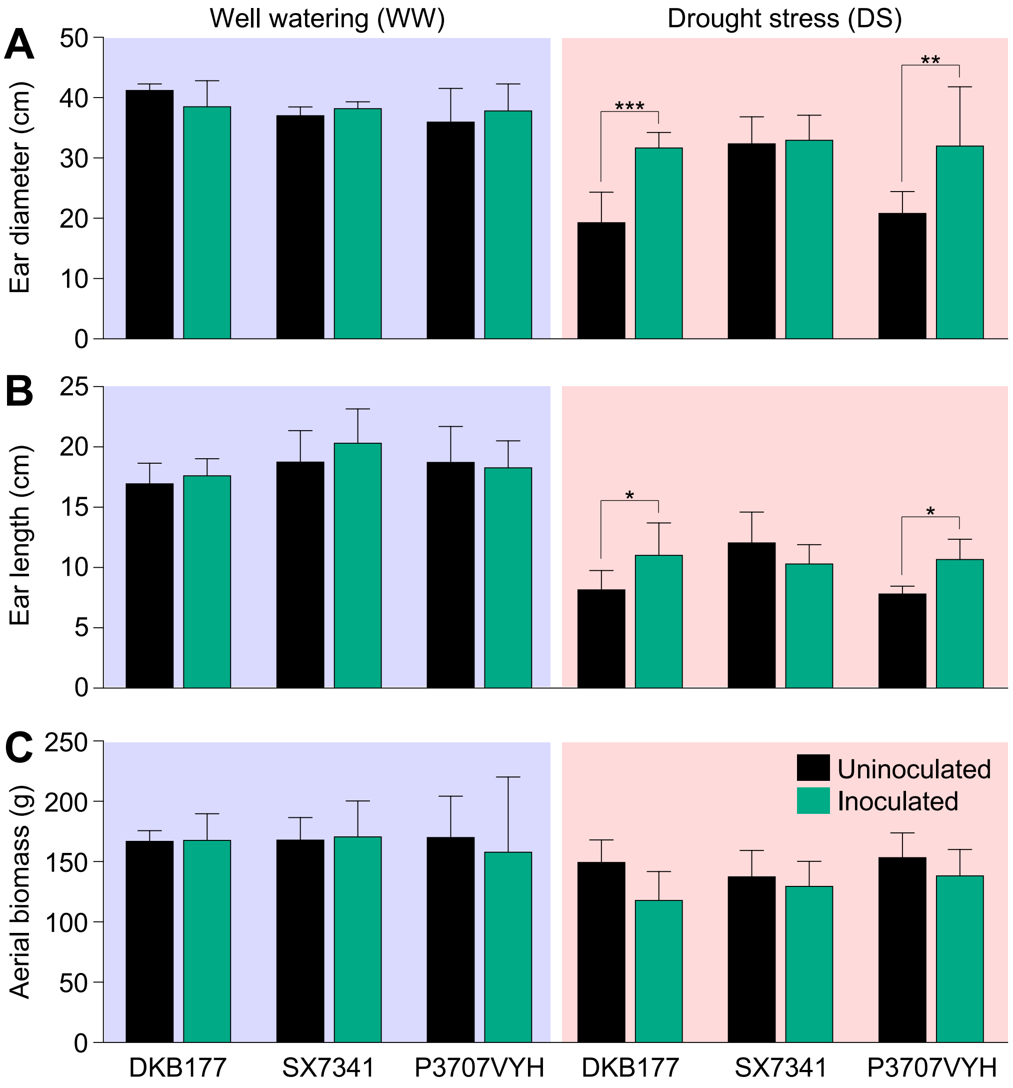

SUPPLEMENTARY FIGURE 5 | SynCom differentially impacts yield-related parameters and plant biomass of commercial hybrids of maize under DS conditions. **(A)** DS-treated inoculated DKB177 and P3707VYH plants had increases of 64 and 54% in ear diameter, respectively, compared to control plants. SX7341 also tended to have an increased ear diameter. **(B)** Under DS, DKB177 and P3707VYH were found to be positively affected with increases of 35 and 36% in ear diameter, respectively, when inoculated. **(C)** No significant difference was found in the biomass of mature plants caused by SynCom, although aerial biomass tended to be higher in WW-treated plants and lower in DS-treated inoculated plants. The main results are shown in **Figure 2**. WW, well watering; DS, drought stress. *$P \leq 0.05$, **$P \leq 0.01$, and *** $P \leq 0.001$.
