# Supplementary material for: Modulating Drought Stress Response of Maize by a Synthetic Bacterial Community
Source: Front Microbiol. 2021 Oct 21;12:747541. doi: 10.3389/fmicb.2021.747541 (PMC8566980; doi:10.3389/fmicb.2021.747541)
Supplement: Supplementary Figure 6 — Maize canopy structure throughout a day as a function of air temperature detected by the non-invasive real-time phenotyping platform. [file Image_6.pdf]

doi: 10.3389/fmicb.2021.747541

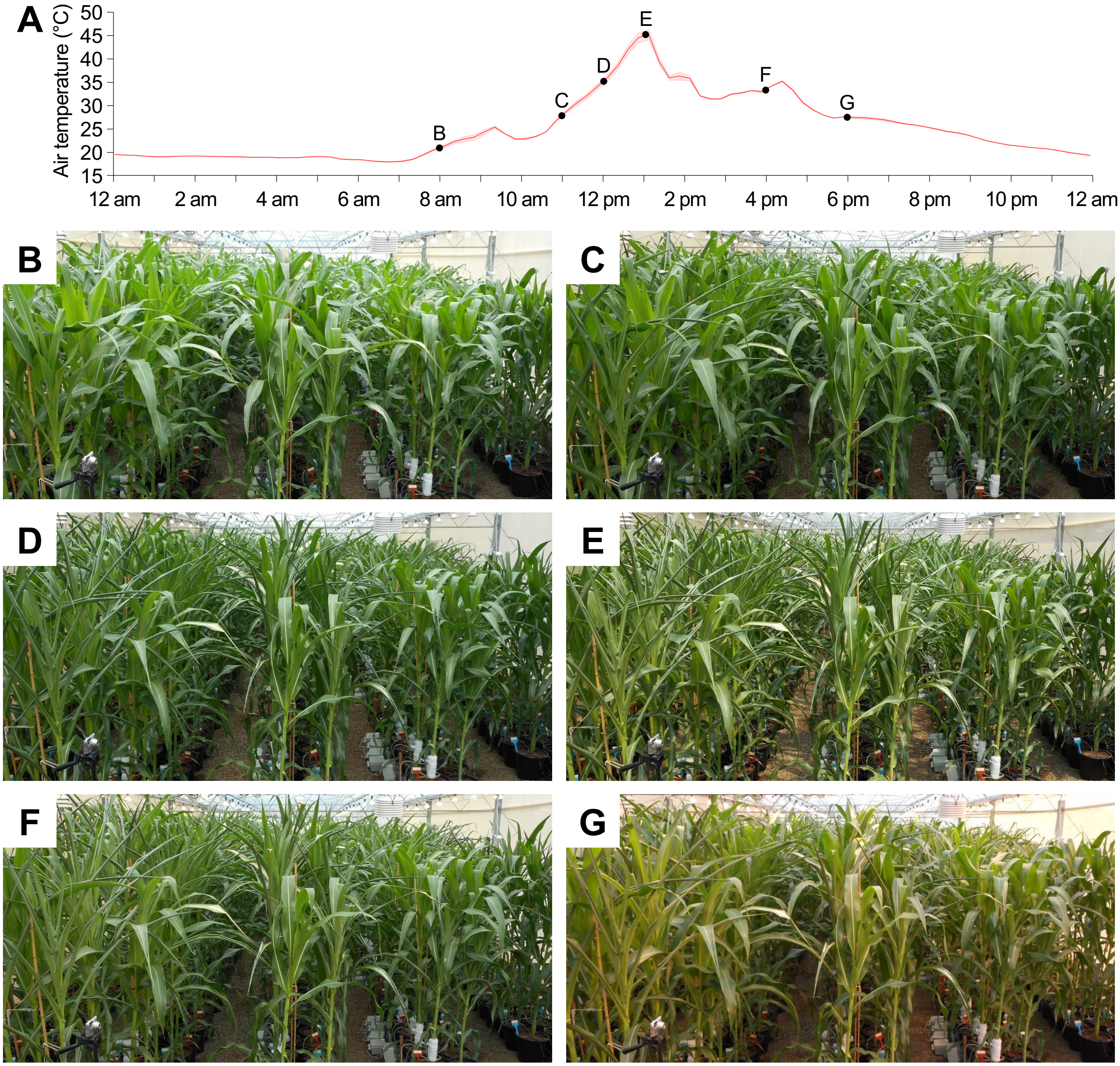

SUPPLEMENTARY FIGURE 6 | Maize canopy structure throughout a day as a function of air temperature detected by the non-invasive real-time phenotyping platform. **(A)** Fluctuation of air temperature throughout the 52nd DAS directly reflected in canopy appearance as shown at **(B)** 8:00 am (21.35±0.35°C), **(C)** 11:00 am (28.90±0.30°C), **(D)** 12:00 pm (36.15±0.65°C), **(E)** 1:00 pm (45.35±1.05°C), **(F)** 4:00 pm (34.35±0.05°C), and **(G)** 6:00 pm (27.55±0.25°C). Plants were, on average, directly affected and had their leaves rolled inward and then straightened upright, as observed from panels **C** to **G**, an effect pronounced during the peak in panel **E**. Values are expressed as the mean ± SD. DAS, days after sowing; SD, standard deviation.
