# Supplementary material for: Modulating Drought Stress Response of Maize by a Synthetic Bacterial Community
Source: Front Microbiol. 2021 Oct 21;12:747541. doi: 10.3389/fmicb.2021.747541 (PMC8566980; doi:10.3389/fmicb.2021.747541)
Supplement: Supplementary Figure 7 — Continuous plant monitoring revealed a direct influence of natural environmental variables in canopy aspect. [file Image_7.pdf]

doi: 10.3389/fmicb.2021.747541

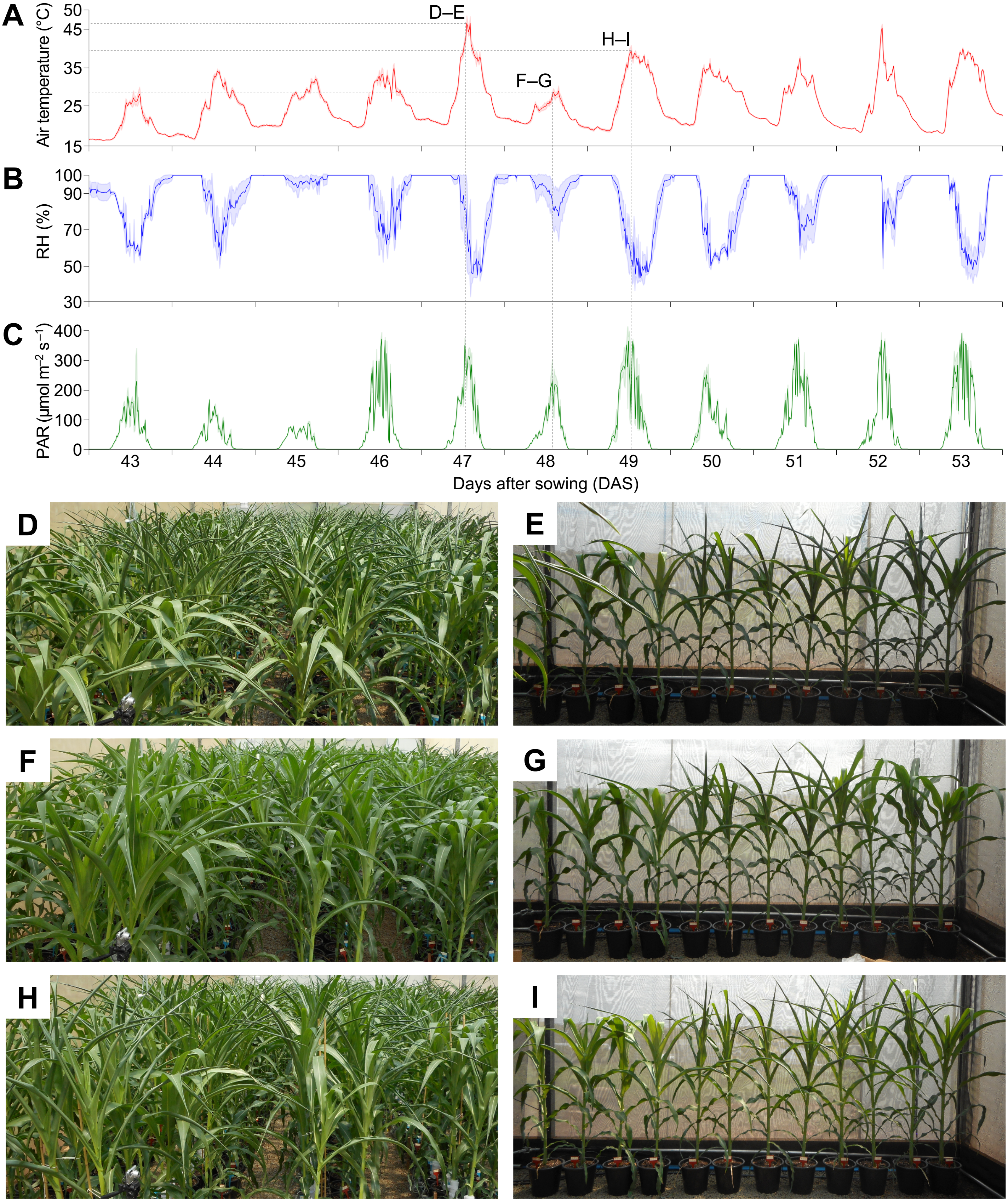

SUPPLEMENTARY FIGURE 7 | Continuous plant monitoring revealed a direct influence of natural environmental variables in the experiment canopy aspect. Fluctuations of air temperature **(A)**, RH **(B)**, and PAR **(C)** were registered by the real-time monitoring platform. Averages per datapoint and SD are shown by solid lines and the colored background, respectively. RGB images were taken from the whole experimental setup **(D,F,H)** and the subset of plants grown for the time-lapse movie **(E,G,I)**. **(D,E)** Nearly all plant leaves rolled inward in an extreme peak of air temperature found at 47 DAS at 1:45 pm (45.03±1.58°C), combined with intermediate RH (64.03±17.91%) and high PAR (321.71±29.45 µmol m^{-2} s^{-1}). **(F,G)** A contrasting event was observed at 48 DAS, when the maximum air temperature reached 28.58±0.98°C at 2:00 pm. Together with higher RH (81.1±11.96%) and relatively lower PAR (147.85±32.87 µmol m^{-2} s^{-1}), plants presented unrolled leaves on average. **(H,I)** An intermediate event was found at 49 DAS at 12:30 pm, when an intermediate temperature (39.4±1.43°C) was observed, combined with lower RH (52.13±3.05%) and reduced PAR (105.26±18.85 µmol m^{-2} s^{-1}), leading plants to express an intermediate phenotype of leaf rolling. Values expressed as the mean ± SD. RH, air relative humidity; PAR, photosynthetically active radiation; SD, standard deviation; DAS, days after sowing.
