# Supplementary material for: Modulating Drought Stress Response of Maize by a Synthetic Bacterial Community
Source: Front Microbiol. 2021 Oct 21;12:747541. doi: 10.3389/fmicb.2021.747541 (PMC8566980; doi:10.3389/fmicb.2021.747541)
Supplement: Supplementary Figure 8 — SynCom differentially affects maize hybrids by dramatically changing Tleaf control under WW conditions. [file Image_8.pdf]

doi: 10.3389/fmicb.2021.747541

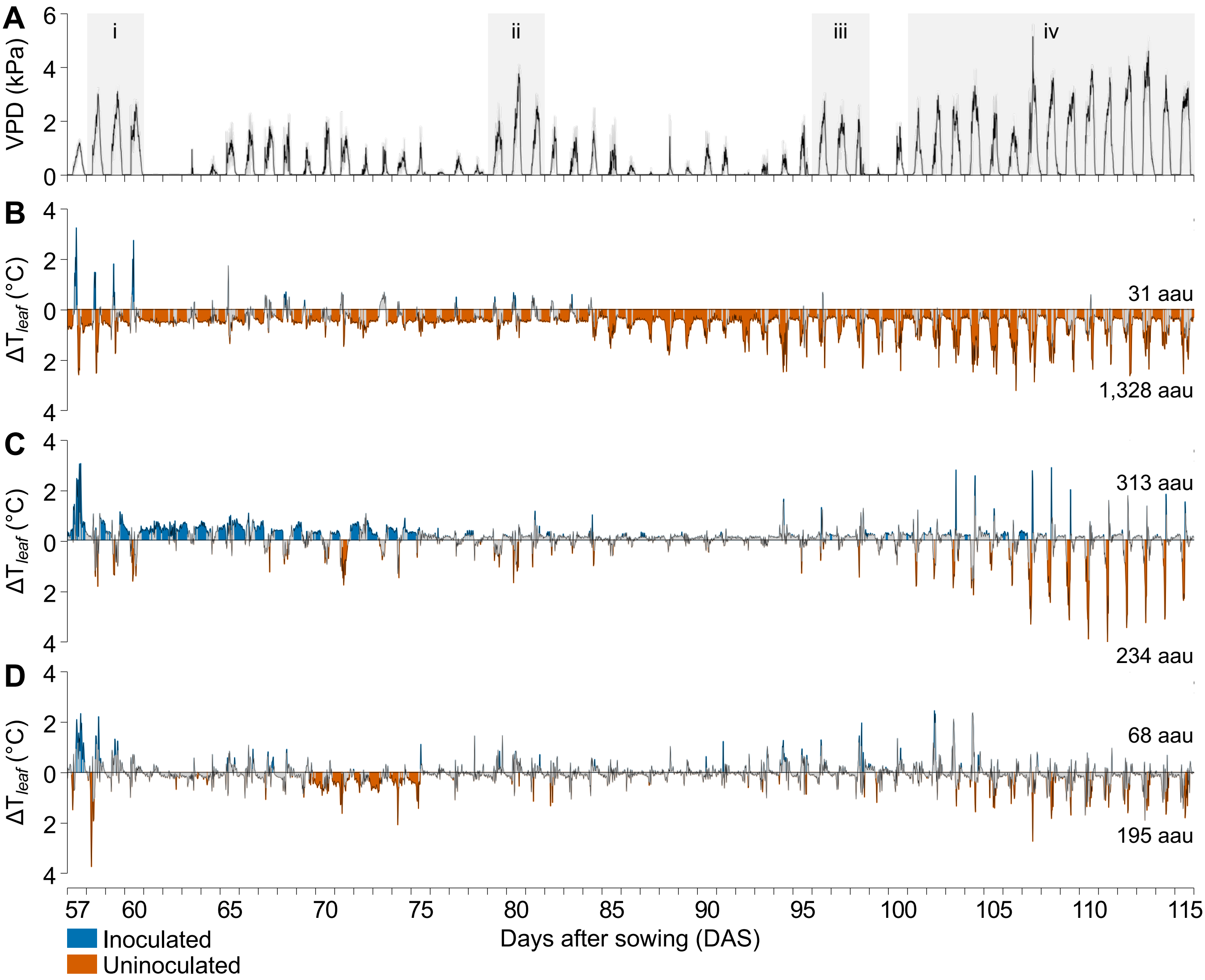

SUPPLEMENTARY FIGURE 8 | SynCom differentially affects maize hybrids by dramatically changing T_{leaf} control under WW conditions. **(A)** Fluctuation of VPD (kPa) revealed tremendous variance throughout 57–115 DAS and a strong influence on T_{leaf}. The gray background highlights days when VPD exceeded 2 kPa (58–60, 79–81, 96–98, 101–115 DAS). **(B–D)** The differences between T_{leaf} of inoculated and uninoculated plants (ΔT_{leaf}), rounded every 30 min over time, revealed that SynCom differentially changed the plant temperature control among all hybrids under WW conditions. Values were displayed above the *x*-axis when T_{leaf} of inoculated plants is higher than T_{leaf} of uninoculated plants or below the *x*-axis when T_{leaf} of uninoculated plants is higher than T_{leaf} of inoculated plants, and colored in blue or red, respectively, when significantly different ($P \leq 0.05$). Areas filled with light gray denote not statistically significant differences. The sums of areas in the graph above and below the *x*-axis were considered only for statistically significant differences. **(B)** DKB177 exhibited a dramatic response to SynCom, as inoculated plants predominantly displayed lower T_{leaf} than uninoculated plants (31 against 1,328 aau, respectively). **(C)** Although the whole period showed an increase in T_{leaf} for SX7341 (313 versus 234 aau for inoculated and uninoculated plants, respectively), during periods with high VPD (> 2 kPa), peaks in T_{leaf} of inoculated SX7341 were overcome by those of SynCom-treated plants. **(D)** Inoculated P3707VYH were, on average, cooler (68 aau) than uninoculated P3707VYH (195 aau), especially when VPD was high. WW, well watering; T_{leaf}, leaf temperature; VPD, vapor-pressure deficit; ΔT_{leaf}, difference of T_{leaf}; aau, arbitrary area units; DAS, days after sowing.
