# Supplementary material for: Modulating Drought Stress Response of Maize by a Synthetic Bacterial Community
Source: Front Microbiol. 2021 Oct 21;12:747541. doi: 10.3389/fmicb.2021.747541 (PMC8566980; doi:10.3389/fmicb.2021.747541)
Supplement: Supplementary Figure 9 — SynCom delays the Tleaf increase in SX7341 maize plants under WW conditions. [file Image_9.pdf]

doi: 10.3389/fmicb.2021.747541

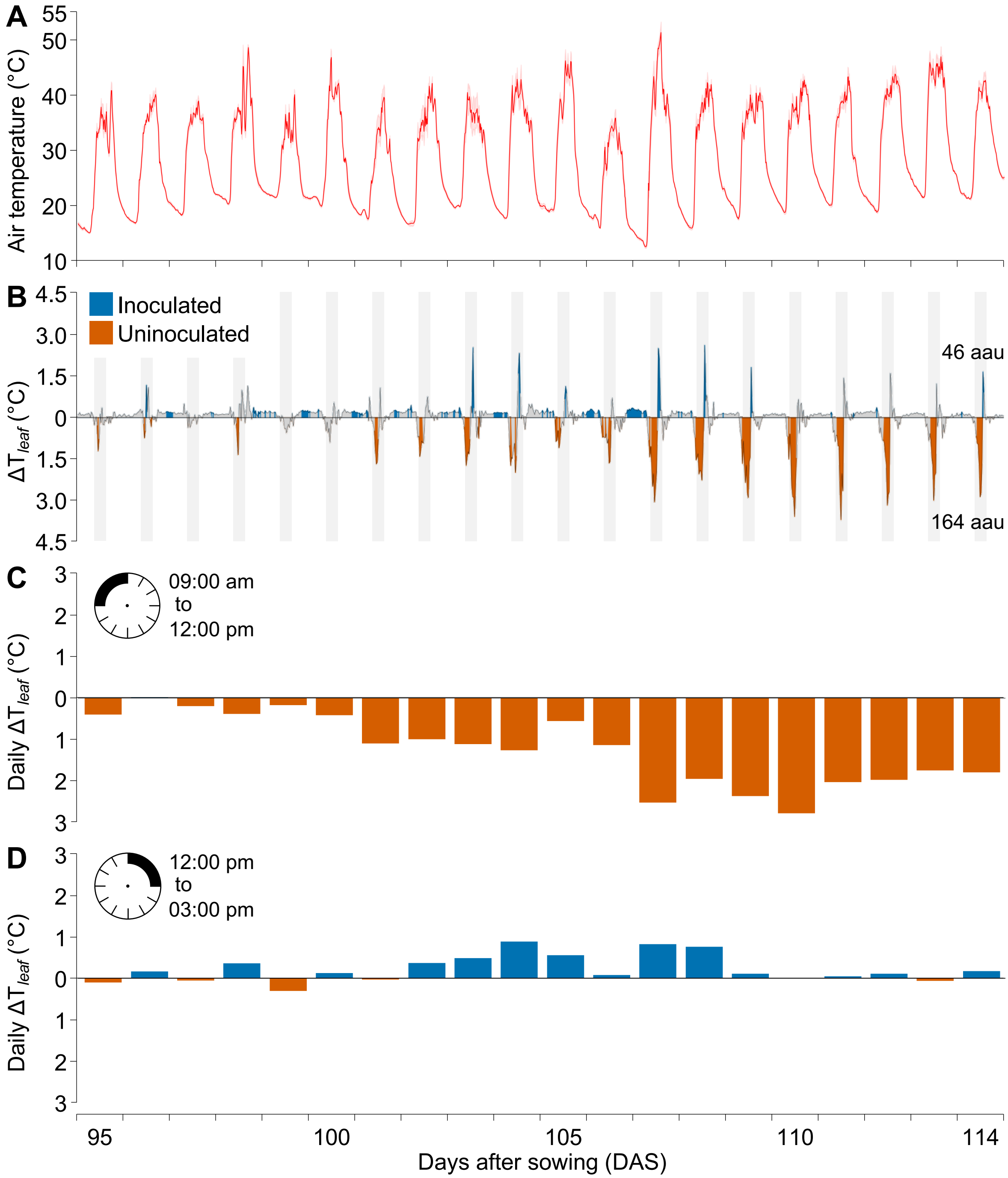

SUPPLEMENTARY FIGURE 9 | SynCom delays the T_{leaf} increase in SX7341 maize plants under WW conditions. **(A)** Fluctuation of air temperature detected by the real-time phenotyping platform from 95–114 DAS. **(B)** The differences in T_{leaf} between inoculated and uninoculated plants (ΔT_{leaf}) rounded every 30 min over time, revealed that the T_{leaf} of inoculated plants was, in general, overcome by the T_{leaf} of inoculated plants (46 against 4,164 aau, respectively). Values were displayed above the *x*-axis when T_{leaf} of inoculated plants is higher than T_{leaf} of uninoculated ones or below the *x*-axis when T_{leaf} of uninoculated plants is higher than T_{leaf} of inoculated ones, and colored in blue or red, respectively, when significantly different ($P \leq 0.05$). Areas filled with light gray denote not statistically significant differences. The sums of areas in the graph above and below the *x*-axis were considered only for statistically significant differences. The gray background highlights daily windows from 9:00 am to 3:00 pm, as shown in panels **C** and **D**. **(C)** In daily periods from 9:00 am to 12:00 pm, the T_{leaf} of inoculated plants was overcome by the T_{leaf} of uninoculated plants. **(D)** In contrast, in the subsequent daily periods, from 12:00 pm to 3:00 pm, the T_{leaf} of the inoculated plants was found to be higher than the T_{leaf} in uninoculated plants. WW, well watering; T_{leaf}, leaf temperature; ΔT_{leaf}, difference of T_{leaf}; aau, arbitrary area units; DAS, days after sowing.
