# Supplementary material for: Modulating Drought Stress Response of Maize by a Synthetic Bacterial Community
Source: Front Microbiol. 2021 Oct 21;12:747541. doi: 10.3389/fmicb.2021.747541 (PMC8566980; doi:10.3389/fmicb.2021.747541)
Supplement: Supplementary Figure 10 — SynCom induces an increase in the Tleaf of maize plants under DS conditions. [file Image_10.pdf]

doi: 10.3389/fmicb.2021.747541

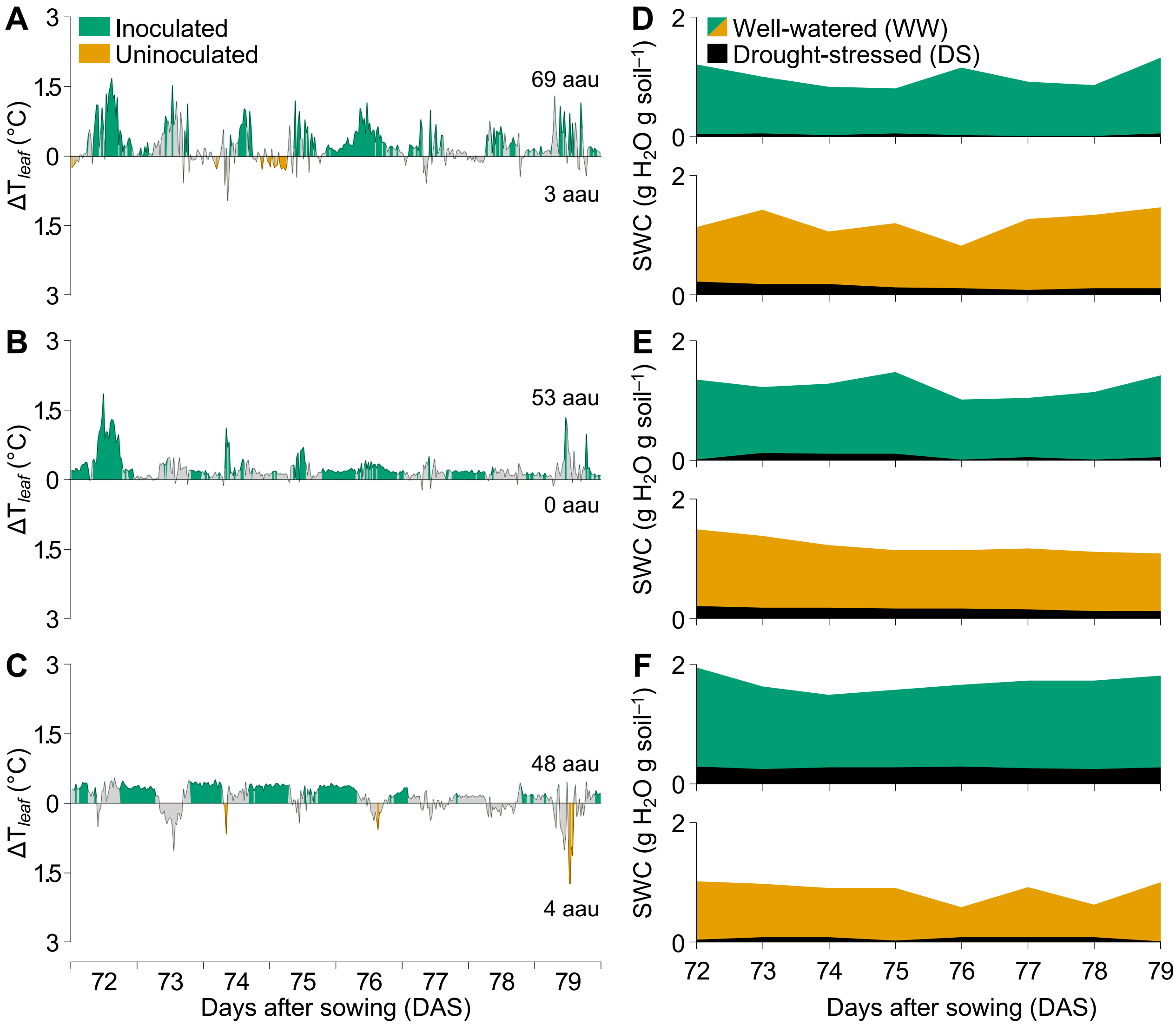


**SUPPLEMENTARY FIGURE 10 |** SynCom induces an increase in the $T_{leaf}$ of maize plants under DS conditions. **(A–C)** The differences in $T_{leaf}$ between inoculated and uninoculated plants ($\Delta T_{leaf}$), rounded every 30 min over time, revealed that SynCom increased $T_{leaf}$ among all hybrids under DS conditions for 72–79 DAS immediately before rehydration. Values were displayed above the *x*-axis when $T_{leaf}$ of inoculated plants was higher than $T_{leaf}$ of uninoculated plants or below the *x*-axis when $T_{leaf}$ of uninoculated plants is higher than $T_{leaf}$ of inoculated plants, and colored in blue or red, respectively, when significantly different ($P \leq 0.05$). Areas filled with light gray denote not statistically significant differences. The sums of areas in the graph above and below the *x*-axis were considered only for statistically significant differences. The exhibited period represents a window showing that $T_{leaf}$ of inoculated DKB177 **(A)**, SX7341 **(B)**, and P3707VYH **(C)** had a significant increase in $T_{leaf}$ when compared with the control plants, as shown by colored areas in the graph. **(D–F)** Measures of SWC showed that inoculated (green) or uninoculated (orange) WW-treated plants were maintained with a high soil moisture when compared with those under DS (black), as shown for DKB177 **(D)**, SX7341 **(E)**, and P3707VYH **(F)**. Due to the large variation in SWC throughout the day, graphs presented the midnight datapoint of each day from 72–79 DAS for $n \leq 4$ pots. SD were omitted since the comparison of every shown WW and DS datapoint was statistically significant ($P \leq 0.001$). $T_{leaf}$, leaf temperature; $\Delta T_{leaf}$, difference of $T_{leaf}$; aau, arbitrary area units; SWC, soil water content; WW, well watering; DS, drought stress; DAS, days after sowing; SD, standard deviation.
