# Supplementary material for: Modulating Drought Stress Response of Maize by a Synthetic Bacterial Community
Source: Front Microbiol. 2021 Oct 21;12:747541. doi: 10.3389/fmicb.2021.747541 (PMC8566980; doi:10.3389/fmicb.2021.747541)
Supplement: Supplementary Figure 11 — Enriched and depleted resident microbial groups in inoculated plants. [file Image_11.pdf]

doi: 10.3389/fmicb.2021.747541

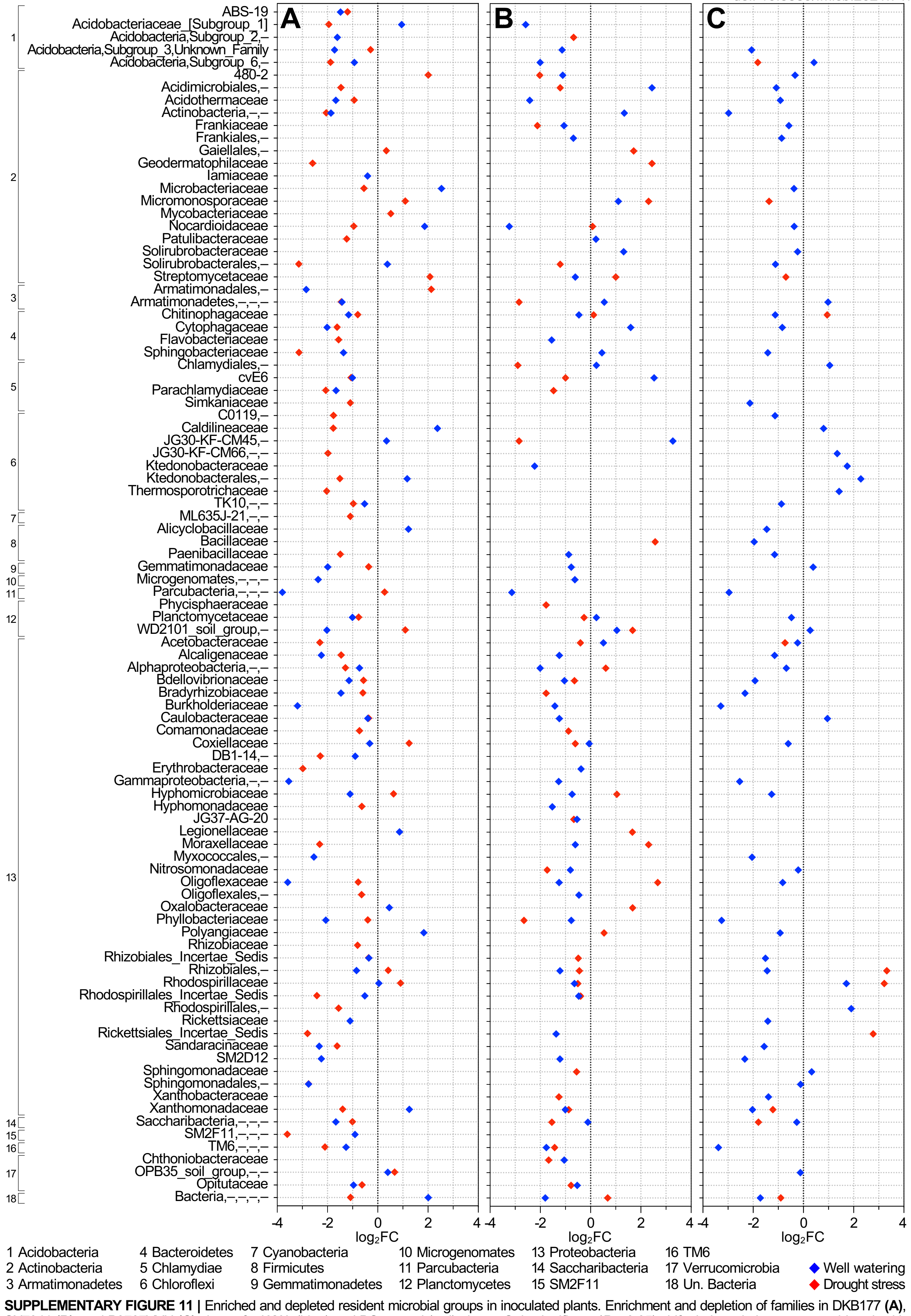

SUPPLEMENTARY FIGURE 11 | Enriched and depleted resident microbial groups in inoculated plants. Enrichment and depletion of families in DKB177 **(A)**, SX7341 **(B)**, and P3707VYH **(C)** shown for WW- (blue) and DS-treated (red) plants. Only significant ($P \leq 0.05$) shifts in inoculation were considered, with OTUs collapsed at the taxonomic level of the family. Families comprised of a single OTU were omitted. "Unknown" taxonomic levels were collapsed to the deepest common levels, comma-separated, and "unknown" taxa represented as "–". FC, fold change; Un., unknown.
