# Supplementary material for: Modulating Drought Stress Response of Maize by a Synthetic Bacterial Community
Source: Front Microbiol. 2021 Oct 21;12:747541. doi: 10.3389/fmicb.2021.747541 (PMC8566980; doi:10.3389/fmicb.2021.747541)
Supplement: Supplementary Table 1 — Three-way ANOVA for phenotypic parameters (yield per plant, kernels per plant, kernel rows per ear, ear diameter, ear length, and aerial biomass) of genotype, irrigation regime, inoculation and all their possible interactions. [file Table_1.pdf]

SUPPLEMENTARY TABLE 1 | Three-way ANOVA for phenotypic parameters (yield per plant, kernels per plant, kernel rows per ear, ear diameter, ear length, and aerial biomass) of genotype, irrigation regime, inoculation and all their possible interactions. *$P \leq 0.05$, **$P \leq 0.01$, and ***$P \leq 0.001$.

Factor	Yield per plant		Kernels per plant		Kernel rows per ear		Ear diameter		Ear length		Aerial biomass	
Genotype	0.0322	*	0.0011	**	0.0000	***	0.0093	**	0.0076	**	0.8992	
Irrigation regime	0.0000	***	0.0000	***	0.0000	***	0.0000	***	0.0585		0.0000	***
Inoculation	0.1153		0.0277	*	0.2793		0.0000	***	0.0000	***	0.0906	
Genotype × Watering regime	0.0644		0.0011	**	0.0000	***	0.0651		0.3333		0.5253	
Genotype × Inoculation	0.0005	***	0.0709		0.0000	***	0.0001	***	0.1634		0.6675	
Irrigation regime × Inoculation	0.0746		0.0078	**	0.0000	***	0.0000	***	0.3373		0.2371	
Genotype × Irrigation regime × Inoculation	0.9863		0.1572		0.0035	**	0.0019	**	0.0028	**	0.6397	

doi: 10.3389/fmicb.2021.747541
