# Supplementary material for: Modulating Drought Stress Response of Maize by a Synthetic Bacterial Community
Source: Front Microbiol. 2021 Oct 21;12:747541. doi: 10.3389/fmicb.2021.747541 (PMC8566980; doi:10.3389/fmicb.2021.747541)
Supplement: Supplementary Table 2 — Harvest index (HI) for inoculated and uninoculated DKB177, SX7341, and P3707VYH under WW and DS conditions. [file Table_2.pdf]

SUPPLEMENTARY TABLE 2 | Harvest index (HI) for inoculated and uninoculated DKB177, SX7341, and P3707VYH under WW and DS conditions. WW, well watering; DS, drought stress.

		Uninoculated			Inoculated		
		Grain yield (g plant^{-1})	Biological yield (g plant^{-1})	Harvest index (%)	Grain yield (g plant^{-1})	Biological yield (g plant^{-1})	Harvest index (%)
WW	DKB177	90.01	167.34	35.0%	101.25	180.16	36.0%
	SX7341	108.48	168.27	39.2%	85.99	170.94	33.5%
	P3707VYH	65.45	187.45	25.9%	81.09	174.16	31.8%
DS	DKB177	9.84	149.73	6.2%	38.72	118.19	24.7%
	SX7341	38.50	137.82	21.8%	30.51	129.98	19.0%
	P3707VYH	12.38	153.83	7.5%	42.65	138.56	23.5%

doi: 10.3389/fmicb.2021.747541
