# Supplementary material for: Modulating Drought Stress Response of Maize by a Synthetic Bacterial Community
Source: Front Microbiol. 2021 Oct 21;12:747541. doi: 10.3389/fmicb.2021.747541 (PMC8566980; doi:10.3389/fmicb.2021.747541)
Supplement: Supplementary Table 3 — Relative abundance of community-based isolates considered robust colonizers in WW- and DS-treated inoculated DKB177, SX7341, and P3707VYH hybrids. [file Table_3.pdf]

SUPPLEMENTARY TABLE 3 | Relative abundance of community-based isolates considered robust colonizers in WW- and DS-treated inoculated DKB177, SX7341, and P3707VYH hybrids. WW, well watering; DS, drought stress; Un., unknown. $^{*}P \leq 0.05$, $^{**}P \leq 0.01$, and $^{***}P \leq 0.001$.

Community-based isolates	OTU	Well watering (WW)			Drought stress (DS)		
		DKB177	SX7341	P3707VYH	DKB177	SX7341	P3707VYH
Agrobacterium sp. E09	Otu2018	0.95%	1.55%	0.83%	0.52%	1.50%	1.08% **
	Otu15158	0.01% *	0.01%	0.01% *	0.01% *	0.01% **	0.01% ***
Asticcacaulis sp. F02	Otu22	0.80%	0.59%	0.74%	0.96%	0.61%	0.77%
Burkholderia sp. A10	Otu11	1.67%	3.88%	2.62%	4.19%	1.61%	2.55%
	Otu199	0.18%	0.41%	0.42%	0.53%	0.08%	0.21%
	Otu17761	0.19% ***	0.13% ***	0.10% *	0.13% *	0.07% *	0.09% ***
Dyella sp. G12	Otu30	0.55%	0.42%	0.62%	0.54%	0.58%	0.66%
Ensifer sp. B04	Otu114	0.86% *	0.79%	0.78% *	0.87% **	0.72%	0.81% ***
Enterobacter sp. B02	Otu18	0.39%	0.78%	0.62%	0.75% **	0.85% ***	0.62%
	Otu186	0.06% *	0.16%	0.11%	0.05% ***	0.04%	0.05% **
Lysobacter sp. A02	Otu9	0.13% **	0.10%	0.09%	0.10% **	0.06% ***	0.11% **
Microbacterium sp. C05	Otu3200	0.03%	0.05%	0.03%	0.02%	0.04%	0.03%
Pantoea sp. B02/C12	Otu16006	0.15% **	0.10% *	0.13% ***	0.13% *	0.13% ***	0.14% ***
Pedobacter sp. A01	Otu29	0.15% **	0.08% *	0.08% **	0.10% **	0.08% **	0.07% **
Sphingomonas sp. D05	Otu255	0.53%	0.48%	0.55%	0.50%	0.58%	0.47%
	Otu16330	0.00% ***	0.00%	0.00% **	0.00% *	0.00% **	0.00% **
Stenotrophomonas sp. E09	Otu20	0.19% *	0.09%	0.11% ***	0.08% *	0.06%	0.13% *
Streptomyces sp. G01	Otu1098	0.37% *	0.45% ***	0.49% ***	0.31% **	0.51% ***	0.34% ***
	Otu8216	0.43% **	0.45% *	0.31%	0.41%	0.24% *	0.29% *
Un. Bradyrhizobiaceae C05	Otu64	0.23%	0.24%	0.22%	0.11%	0.26%	0.25%
Un. Xanthomonadaceae B08	Otu84	0.44% **	0.25%	0.20% *	0.49% **	0.23% *	0.23% **
Un. Xanthomonadaceae G08	Otu12	0.43% **	0.39% *	0.47% **	0.47% *	0.30% *	0.32% **
Variovorax sp. F04	Otu45	0.49%	0.43%	0.41% *	0.45% **	0.46%	0.42%

doi: 10.3389/fmicb.2021.747541
